# Activity of ice-binding proteins can be markedly enhanced by protein tags

**DOI:** 10.1039/d5nr04805b

**Published:** 2026-03-11

**Authors:** Daniëlle van den Broek, Sanne N. Giezen, Tim P. Hogervorst, Renko de Vries, Ilja K. Voets

**Affiliations:** a Laboratory of Self-Organizing Soft Matter, Department of Chemical Engineering and Chemistry and Institute for Complex Molecular Systems, Eindhoven University of Technology 5600 MB Eindhoven The Netherlands i.voets@tue.nl; b Department of Physical Chemistry and Soft Matter, Wageningen University and Research 6708 WE Wageningen The Netherlands

## Abstract

Ice-binding proteins (IBPs) are crucial for the survival of cold-adapted organisms, as they regulate ice crystal formation and growth. To understand their molecular mode of action, fluorescence microscopy of IBPs bound to ice-crystal surfaces has been shown to be very helpful, although it is unknown whether the (fluorescent) tags typically used in these studies affect the activities of the IBPs. Here, we evaluate the impact of mEos3.2, SNAP-tag, and HaloTag on the ice-recrystallization inhibition (IRI) activity of IBPs. We find that most tags, in most orientations, do not affect the IRI activity of IBPs. These tags are promising candidates for investigating the binding mechanisms of IBPs in their native form with fluorescence microscopy. A surprising exception is the N-terminal attachment of HaloTag to QAE, an isoform of AFP type III: for this case, we find an order of magnitude higher IRI activity. Additionally, we show that HaloTag also has moderate IRI activity by itself and induces the formation of ice crystals with hexagonal prism morphology, suggesting binding affinity for the primary prism plane of ice. Our findings indicate that moderately IRI-active proteins may synergistically enhance the IRI activity of IBPs, when attached in the proper orientation.

## Abstract 2

Ice-binding proteins (IBPs) regulate ice crystal growth, in particular through ice recrystallization inhibition (IRI). IRI activity can be synergistically enhanced when IBPs are fused to protein tags with moderate activity.

## Graphical abstract

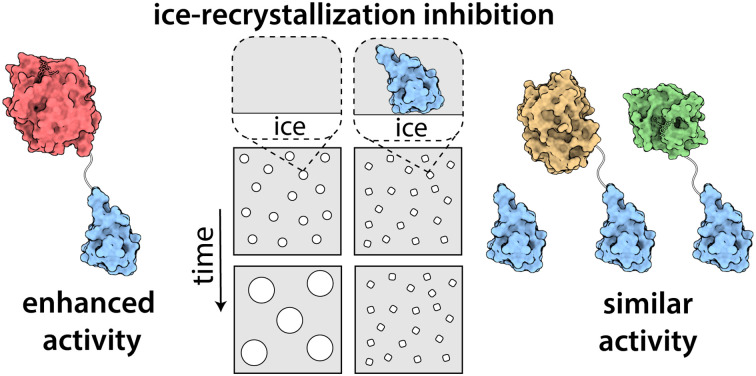

## Introduction

Ice-binding proteins (IBPs) are produced by organisms in cold habitats to control the formation and growth of ice crystals.^1^ These proteins inspire the development of ice-binding materials that are optimized to mitigate freezing damage in societal applications,^2^ including cryopreservation,^3^ frozen food storage, and agriculture.^4^ One particular form of freezing damage is caused by ice recrystallization during thawing: small precursor crystallites grow into large crystals, which can damage the structure of frozen food,^5^ cells, and tissues.^6^ This type of damage could potentially be prevented by IBPs with high ice recrystallization inhibition (IRI) activity.^7,8^

**Fig. 1:**
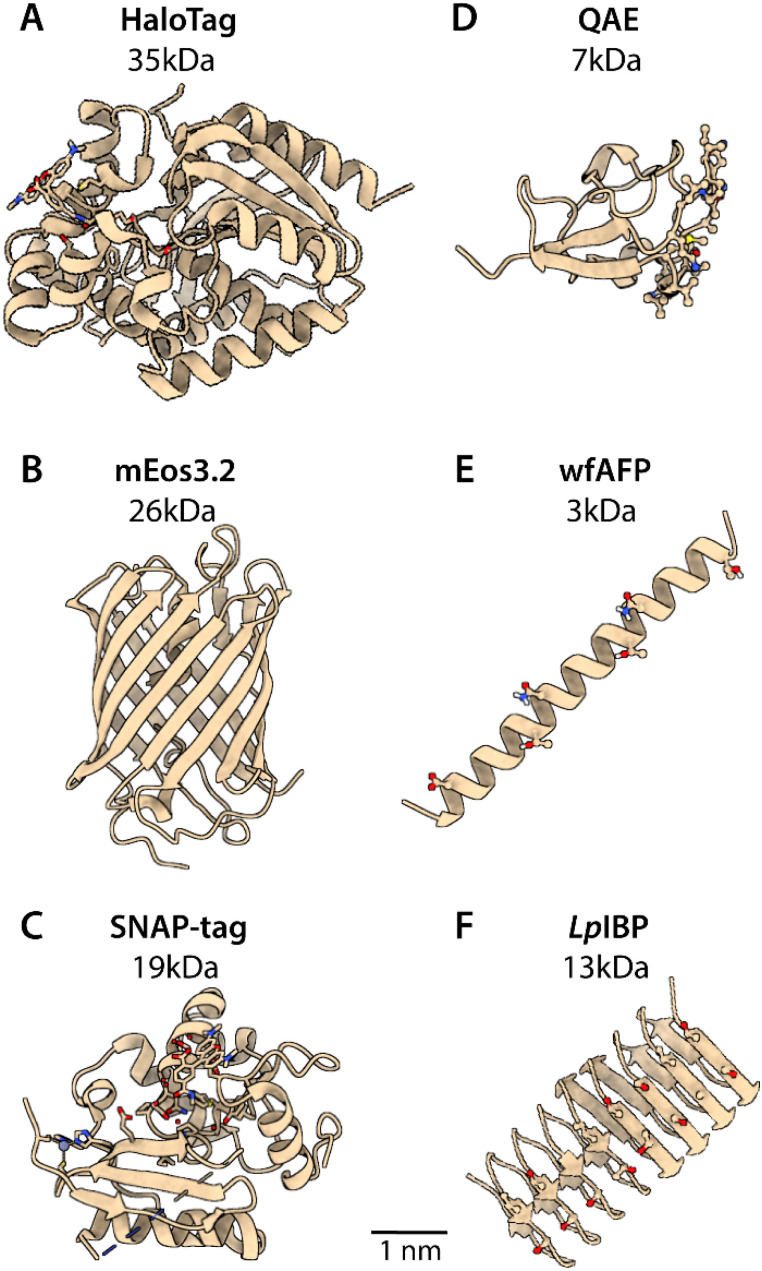
Structures and relative sizes of the protein tags and ice-binding proteins (IBPs) used in this study. (A–C) Three protein tags that are applicable in (super-resolution) fluorescence microscopy. mEos3.2 (PDB: 9J11) contains a photoconvertible fluorophore in its barrel. HaloTag (PDB: 6U32) and SNAP-tag (PDB: 6Y8P) are not fluorescent by themselves, but require the attachment of a synthetic ligand, such as a reactive chloroalkane (HaloTag) or O^6^-benzylguanine linker (SNAP-tag) bound to a (photoconvertible) fluorescent dye. (D–F) Three structurally diverse IBPs: QAE (HPLC12 AFP type III isoform from ocean pout, PDB: 1HG7), wfAFP (from winter flounder, PDB: 1WFA) and *Lp*IBP (from perennial rye grass *Lolium perenne*, PDB: 3ULT).

To obtain IBPs with highly efficient IRI activity, a more profound understanding of the binding mechanisms of IBPs is essential. Previously, we have applied single-molecule localization microscopy (SMLM) to study the dynamics of single IBPs at the ice-water interface.^9^ For such studies, the IBPs have to be labelled with specific tags that facilitate fluorescence imaging, raising the question whether these tags can influence activities such as IRI. Effects of tags or fusion partners on activities of ice-binding proteins have been reported before for another type of activity of ice-binding proteins, namely thermal hysteresis (TH) activity. For instance, the TH activity of QAE – an isoform of AFP type III – was found to be moderately enhanced by the attachment of thioredoxin or maltose-binding protein. Presumably, this is the case because engulfment of the protein by ice is less likely to occur for a protein with a larger non-ice-binding site.^10,11^ Increasing the size of the ice-binding site (IBS) through dimerization also caused a moderate increase in TH activity for AFP III^12^ and IBPv (from an Antarctic bacterium in the *Flavobacteriaceae* family).^13,14^ It has also been demonstrated that in mixtures of IBPs with affinity for different ice crystal planes, there may be synergistic enhancement of TH activity.^15^ In contrast, for IRI activity of IBPs, very few interaction effects have been reported. Moderately synergistic effects on IRI activity have been observed for combinations of AFP I, AFP III, and AFGP,^16^ and for AFP III in mixtures with various food biopolymers.^7,16,17^ We are not aware of any previous work on the impact of protein tags on the IRI activity of IBPs. Given the demonstrated influence of various protein tags on TH activity and the large size of typical protein tags compared to IBPs (Fig. 1), we hypothesized that the IRI activity of IBPs could also be influenced by the attachment of a protein tag; *i.e.* there could be synergistic or antagonistic interactions between the tag and IBP with respect to IRI activity.

Here, we examine whether protein tags used for fluorescence microscopy affect IRI activity. During our studies, we discovered that HaloTag, a self-labeling protein tag typically used for covalently attaching fluorescent dyes to proteins,^18,19^ markedly increases IRI activity of QAE (the HPLC12 isoform from ocean pout *M. americanus*) when attached N-terminally. Additionally, we found that it has an appreciable IRI-activity by itself. This surprising case is investigated in considerable detail. We first quantify IRI activities of QAE, *Lp*IBP^20–22^ (from the grass *Lolium perenne*) and wfAFP^23,24^ (from winter flounder), both untagged and fused to various tags (HaloTag,^18,19^ mEos3.2^25^ or SNAP-tag^19,26^). Next, we demonstrate conditions under which the HaloTag itself exhibits IRI activity, or enhances IRI activity of IBPs. Finally, we show how the HaloTag itself, and its fusion to IBPs affects the shaping of ice crystals.

## Experimental section

### Gene construction

The pET28a(+) expression vectors with inserts that encode mEos3.2-QAE, mEos3.2-QAE(T18N), HaloTag-QAE, HaloTag-QAE(T18N) and HaloTag-wfAFP were constructed by as previously described.^9^ The same method was applied to generate the pET28a(+) vector with HaloTag-QAE(A16R) insert.

For *Lp*IBP, a DNA fragment (geneblock) flanked by NcoI/XhoI restriction sites was purchased at IDT. The DNA fragments for QAE, QAE(T18N) and QAE(A16R) with NcoI/XhoI restriction sites were PCR amplified from the constructs listed above. The DNA fragments were cloned into NcoI/XhoI restricted pET28a(+) vectors. All constructs were sequence verified with Sanger sequencing. For expression of HaloTag, QAE-HaloTag, HaloTag-*Lp*IBP, HaloTag-SUMO and SNAPtag-QAE, the pET28a(+) vectors with corresponding inserts were purchasezd at Twist Bioscience (clonal genes: Twist Bioscience cloned the DNA fragments in between NdeI/XhoI restriction sites of pET28a(+) vectors). The sequences of all protein constructs can be found in Table S1.

### Protein expression and purification

The pET28a(+) expression vectors encoding for the recombinant proteins were transformed into *E. coli* BL21(DE3) and spread on LB agar plates containing kanamycin (50 µg mL^−1^). One colony was used to inoculate 8 mL LB medium with kanamycin. Small cultures were incubating overnight while shaking at 37 °C, and transferred to 1 L LB medium with kanamycin and incubated until the OD reached 0.7–0.8. Then protein expression was induced by adding IPTG to a final concentration of 1 mM, and the cultures were incubated at 18 °C for 20 hours while shaking at 150 rpm. The cultures were centrifuged at 10.000*g* for 10 min at 4 °C, and the cells were resuspended in cold lysis buffer (50 mM TRIS, 300 mM NaCl, pH = 7.5) supplemented with lysozyme and EDTA-free protease inhibitor cocktail (Sigma). Then the cells lysed by sonication on ice and the lysate was centrifugated at 4.000*g* for 1 hour at 4 °C. Ni–NTA agarose HIS-bind resin (Qiagen) with a volume of ∼2 mL in a disposable gravity columns (Biorad) was equilibrated with cold buffer (50 mM TRIS, 300 mM NaCl, pH = 7.5), and the supernatant was applied onto the columns. The resin was washed with 10 column volumes of cold washing buffer (50 mM TRIS, 300 mM NaCl, 10–20 mM imidazole, pH = 7.5). The proteins were eluted from the columns with elution buffer (50 mM TRIS, 300 mM NaCl, 400 mM imidazole, pH = 7.5). Next the buffer was exchanged to a protein storage buffer (20 mM TRIS pH = 7.5 for all proteins, except HaloTag-wfAFP for which PBS pH = 7.5 was used) with PD-10 desalting columns (Cytvia). Protein purity was evaluated by running SDS-PAGE gels (Fig. S6). Protein concentrations were determined by measuring absorbance at 280 nm with a Nanodrop1000 instrument, or with SDS-PAGE gel band analysis in comparison to a BSA standard. All proteins were supplemented with 10%v/v glycerol, flash frozen in liquid nitrogen, stored at −70 °C and thawed on ice before use. Additional information regarding the reproducibility and purity of protein constructs with HaloTag is in the SI (page 13).

### Solid phase peptide synthesis

The peptides wfAFP^23,24^ and AFGP (AAT^d^AAT^d^AAT^d^AAT^d^AA: a synthetic analogue of the natural AFGP8^27–29^) were produced *via* solid phase peptide synthesis (methods in SI (page 21)).

### Ice recrystallization inhibition (IRI)

Square coverglasses (Epredia, #1.5, 22 × 22 mm) and round coverglasses (VWR, #1, 15 mm diameter) were cleaned by sonication for (5 minutes in MQ and 5 minutes in acetone) and dried under N_2_ gas flow. Protein samples with the desired concentrations were prepared by diluting in buffer (20 mM TRIS or PBS pH = 7.5) and buffer with 60 wt% sucrose to a final sucrose content of 40 wt%. 0.9 μL of sample was applied onto a square coverglass and sandwiched by a round coverglass. Imaging was performed on a Nikon ECLIPSE Ci-Pol optical microscope equipped with a Linkam LTS420 stage. The sample was frozen to −40.0 °C at 20 °C min^−1^, heated to −10.0 °C at 10 °C min^−1^, followed by heating to −8.0 °C at 1 °C min^−1^. Then the temperature was held constant for 60 minutes, and ice-recrystallization was monitored with a Nikon L Plan 20× (NA 0.45) objective by recording one image every minute.

Ice-recrystallization rates were analyzed with a custom Python script using the PyImageJ package, based on a previously described method: ice recrystallization rate inhibition analysis (IRRINA).^27,30^ In short, the ice crystal images were first subjected to a bandpass filter, contrast enhancement and background subtraction. Subsequently, the AutoThreshold and Convert to Mask functions were used to generate binary images that indicate the crystal edges. The area of each crystal was measured with the Analyze Particles function and used to calculate the radius *r*. Crystals that touched the border of the image or had a circularity below 0.35 were not taken into account. The cubic average radius^3^ was calculated for each time point and plotted as a function of time. The ice-recrystallization rate was determined by applying a linear fit to the data in the 20–60 minutes time window.

### Ice crystal shaping

Square coverglasses (Epredia, #1.5, 22 × 22 mm) and round coverglasses (VWR, #1, 15 mm diameter) were cleaned by sonication for (5 minutes in MQ and 5 minutes in acetone) and dried under N_2_ gas flow. Protein samples with the desired concentrations were prepared by diluting in with buffer (20 mM TRIS pH = 7.5) and buffer with 60 wt% sucrose to a final sucrose content of 30 wt%. Protein concentrations chosen for the ice crystal shaping experiments are the IRRINA assay endpoints: the lowest concentrations that show complete IRI activity in the IRRINA assay. The IRRINA assay endpoints of *Lp*IBP and HaloTag were determined in this work, and the endpoint of the AFGP analogue was adopted from literature.^27^ A more detailed explanation about the chosen concentrations and the application of sucrose is included in the SI (page 7). Tape with a thickness of 50 µm was used to create spacers for ice crystal shaping samples. Circular holes with a diameter of 10 mm were cut into the tape. The tape was transferred onto a square coverglass and 4.5 µL sample was applied onto the glass in the middle of the cut-out area. A round coverglass was used to sandwich the sample, after which the sample was flattened further by pressing on it gently it with a concentric press. The sample was transferred to a Nikon ECLIPSE Ci-Pol optical microscope with a crossed polarizer-analyzer set-up and rotatable microscope table (Fig. S2). A first order wave retardation plate (U-TP530) was inserted in between the objective and the analyzer, orientated 45° with respect to the polarizer and analyzer. With this set-up the orientation of the ice crystals was determined. The Linkam LTS420 stage was used to freeze the sample to −40.0 °C at 20 °C min^−1^. The temperature was increased gradually to −20.0 °C with 20.0 °C min^−1^, to −8.0 °C with 10 °C min^−1^, to −6.0 °C with 1.0 °C min^−1^, to −5.0 °C with 0.5 °C min^−1^ and further with 0.2 °C min^−1^ until stable single crystals could be observed at ±−4.0 °C. Then the crystals were allowed to grow slowly by cooling the sample with 0.2 °C min^−1^. The appearance of ice crystal shaping was monitored in bright field mode with the 20× (NA 0.45) objective by recording an image every 30 seconds. The temperature was held constant after cooling 0.4 °C, when a shaping pattern could be observed. The microscope was switched to polarized light mode by transferring the analyzer into the light path. The white balance was adjusted to the background before imaging. The microscope stage was rotated and an image was recorded at 0°, 30°, 60° and 90° of rotation. The orientation of the *c*-axis (optical axis) of the crystal was deduced from its color and its orientation with respect to the optical axis of the retardation plate (Fig. S3 and S4). More detailed information about this method can be found in the SI (page 3–6). The *c*-axis lengths (*L*_*c*_) and *a*-axis lengths (*L*_*a*_) of multiple individual crystals were measured using ImageJ. *L*_*c*_/*L*_*a*_ axis ratios were calculated for crystals in the presence of *Lp*IBP, AFGP and HaloTag, and statistical analysis was performed to test for significant differences between the three groups (Kruskal–Wallis test and Dunn's multiple comparisons test).

### Protein structures

The protein structures were retrieved from the protein data bank (PDB codes in Table S2) and images were created in UCSF ChimeraX (Fig. 1, 3 and 4). For the mutants QAE mutants, the original amino acid residues from the wild-type QAE were substituted with mutated residues using the most frequently occuring rotaitions form the Dunbrack backbone-dependent rotamer library. Structure models of the fusion constructs were generated using AlphaFold 3 ^31^ (Fig. 8 and Fig. S5).

## Results

### Influence of various protein tags on the ice-recrystallization activity of IBPs

We chose the IBP QAE as a starting point to study the influence of protein tag attachment on IRI activity, as QAE is structurally well-characterized and easy to express.^32–37^ For our studies, we constructed fusions of QAE to relevant tags typically used to generate fluorescently labeled proteins: HaloTag, mEos3.2, and SNAP-tag. In each case, the tag is attached N-terminally and a GAG linker is used between the tag and QAE.

**Fig. 2:**
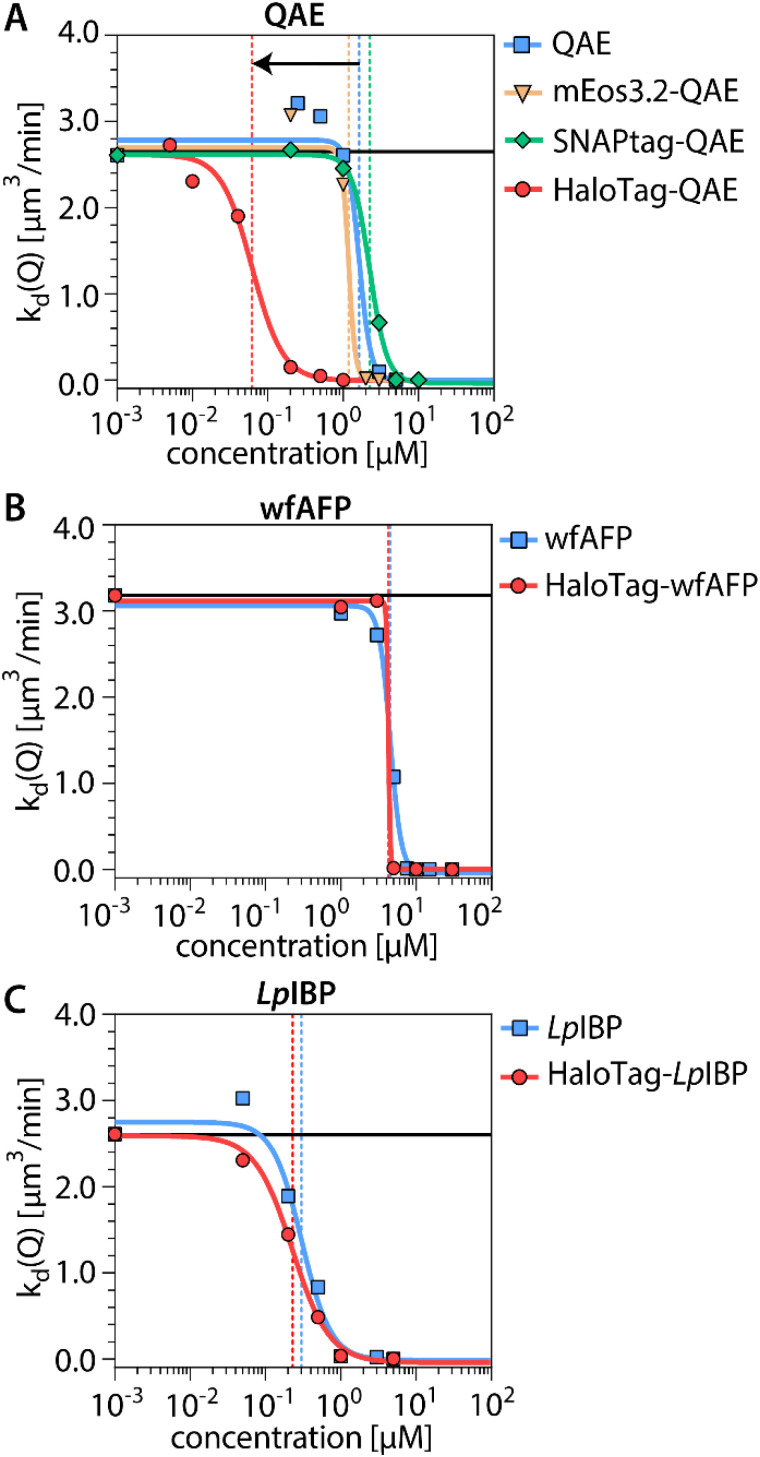
Ice recrystallization inhibition (IRI) activity of three tagged and untagged IBPs. (A) QAE (AFP III) (B) wfAFP and (C) *Lp*IBP. The average crystal recrystallization rate is indicated as a function of protein concentration. The colored vertical lines denote the inhibitory concentration *c*(i) of each protein at the inflection point of the curve. The black horizontal line indicates the recrystallization rate in the absence of IBP.

Ice-recrystallization inhibition (IRI) activities of the recombinant protein constructs were quantified for a range of concentrations in a so-called ice recrystallization rate inhibition analysis (IRRINA) assay,^27^ using bright field microscopy. Fusion of HaloTag to QAE leads to a marked increase in IRI activity of well over one order of magnitude (Fig. 2A), while the mass of the protein increases only five times. On the other hand, attachment of SNAP-tag or mEos3.2 to QAE does not affect IRI activity. In view of the strong enhancement of QAE IRI activity by HaloTag, we wondered whether HaloTag might increase TH activity as well. However, we found that the attachment of HaloTag only leads to a slight increase in the TH activity of QAE (SI). This increase is in fact smaller than the increase we find with mEos3.2 (Fig. S1). Next, we assessed how the IRI activity of two other ice-binding proteins is affected by the attachment of HaloTag: wfAFP (Fig. 2B) and *Lp*IBP (Fig. 2C). The HaloTag is again attached N-terminally, and a GGGS or GGGSGGGS linker is used between the tag and the IBP to avoid steric hindrance. We find that HaloTag does not influence the IRI activity of these two IBPs. Hence, the enhancement appears specific for the combination of QAE with HaloTag.

**Fig. 3:**
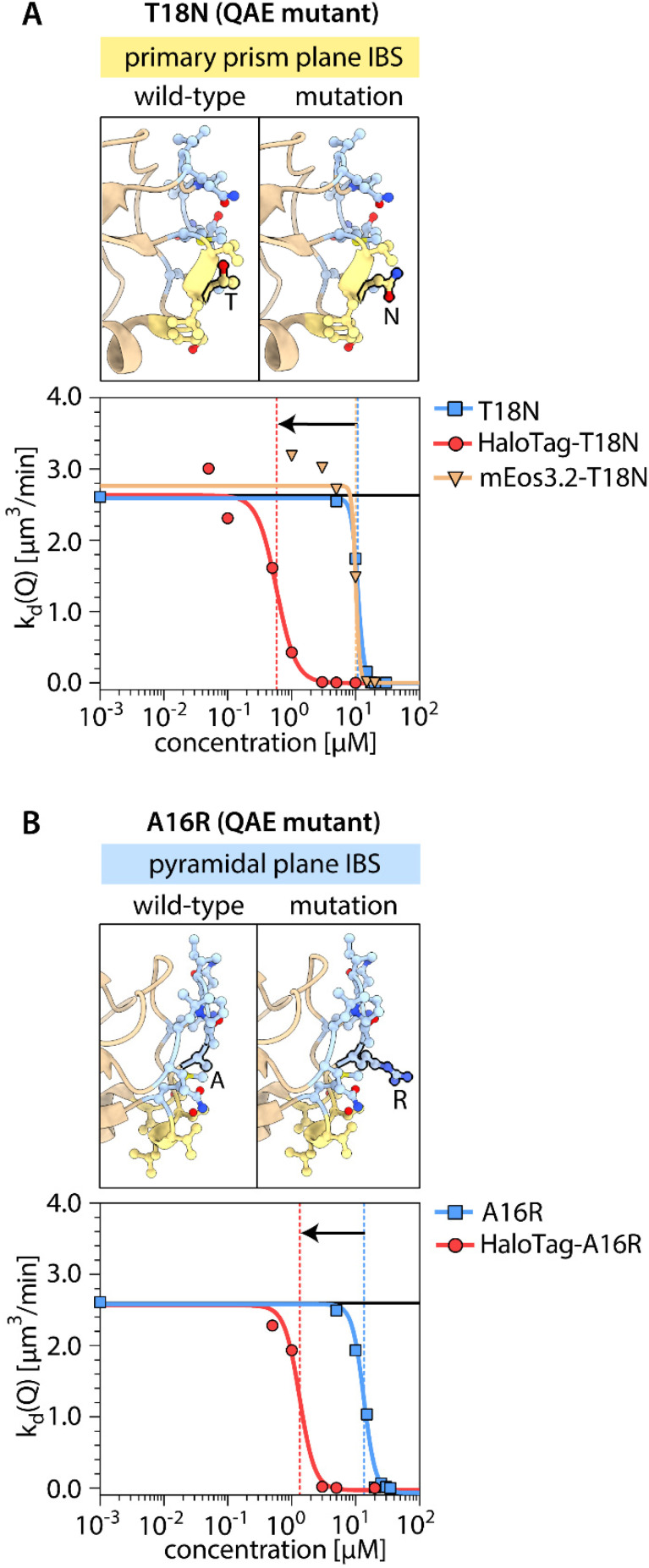
IBS structures and ice recrystallization inhibition (IRI) activities of the QAE wildtype and two mutants with and without HaloTag. (A) T18N with a mutation in the primary prism plane binding site (yellow). (B) A16R with a mutation in the pyramidal plane binding site (blue). The average crystal recrystallization rate is indicated as a function of protein concentration.

**Fig. 4:**
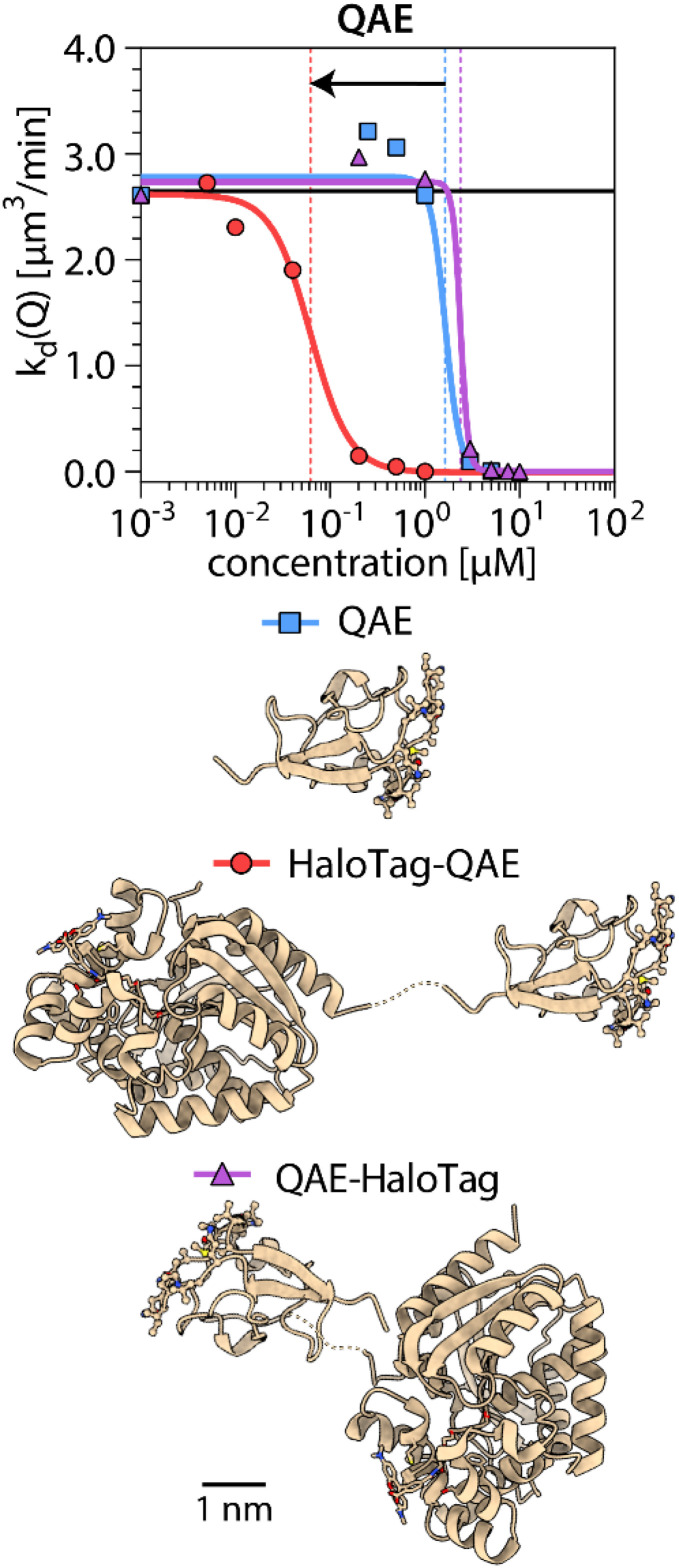
Ice recrystallization inhibition (IRI) activities and protein structures of HaloTag fusion constructs with two linking positions. HaloTag is linked to either the N-terminus (red) or the C-terminus (purple) of QAE. The average crystal recrystallization rate is indicated as a function of protein concentration. The colored vertical lines denote the inhibitory concentration *c*(i) of each protein at the inflection point of the curve. The black horizontal line indicates the recrystallization rate in the absence of ice-binding protein.

**Fig. 5:**
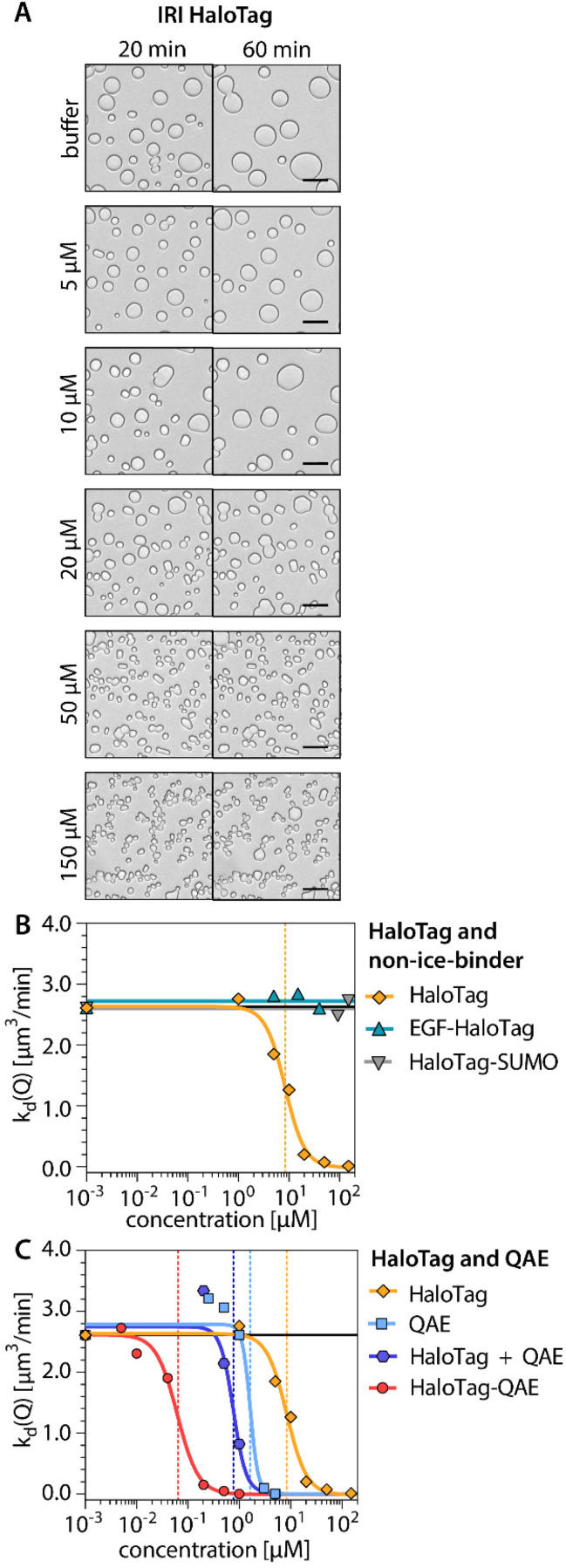
Ice recrystallization inhibition (IRI) activities of the HaloTag and fusion constructs to non-ice-binding proteins. (A) Brightfield microscopy images showing the IRI activity of the independent HaloTag at a range of concentrations. (B and C) The average crystal recrystallization rate is indicated as a function of protein concentration. (B) Recrystallization rates with the HaloTag independently compared to those with fusion constructs of the HaloTag with a non-ice-binding protein (epidermal growth factor (EGF) or SUMO (small ubiquitin like modifier)). (C) Recrystallization rates with separately combined HaloTag and QAE (1 : 1 molar ratio, indicated concentrations refer to the total protein content) compared to those with the fusion construct HaloTag-QAE, HaloTag independently and QAE independently. All scale bars: 20 μm.

Next, we investigated further potential factors that might lead to the enhancement of IRI activity specifically for the combination of QAE and HaloTag. The IBS of QAE is known to be composed of two subsites.^34^ One has affinity for the primary prism plane {100}, and another is reported to have affinity for a pyramidal plane with unspecified Miller indices.^34^ It has been suggested that mutations on either of these subsites remove or reduce the binding affinity of QAE to the corresponding ice-crystal planes.^34^ If such mutations would alter the synergy with the HaloTag, we might be able to infer whether the synergy is related more to primary prism plane binding or to pyramidal plane binding. Therefore, we introduced a point mutation in each region of the QAE IBS: a T18N mutation^36^ in the primary prism plane binding site and an A16R mutation^36^ in the pyramidal plane binding site. We find that IRI activity of both mutants is enhanced by one order of magnitude upon attachment of the HaloTag, while the activity of T18N is not affected by attachment of mEos3.2 (Fig. 3). Another factor that may influence the synergistic enhancement of IRI activity of QAE by HaloTag is whether the HaloTag is attached N- or C-terminally. We found that the IRI activity of QAE with C-terminally attached HaloTag is the same as that of native QAE (Fig. 4). Having found that the enhancement in IRI activity of QAE by HaloTag is quite specific, we wondered whether HaloTag itself might have affinity for ice, which could contribute to the effects that we observe. With this in mind, we monitored ice-recrystallization in the presence of HaloTag itself (not attached to an IBP). Surprisingly, we found that HaloTag inhibits ice recrystallization (Fig. 5A). This raised the question whether this tag could impart IRI activity to constructs with a non-ice-binding protein. Therefore, we determined the IRI activity of fusion proteins of the HaloTag to non-ice-binding proteins with molecular weights comparable to QAE. For HaloTag attached to the N-terminus of EGF^38^ (epidermal growth factor, 7 kDa) and for HaloTag attached to the C-terminus of SUMO^39,40^ (small ubiquitin like modifier, 11 kDa), we find no IRI activity (Fig. 5B). Next, we tested whether HaloTag enhances the IRI activity of QAE when used in a mixture rather than in a fusion construct, and the mixture did not show enhancement in IRI activity (Fig. 5C). In sum, the synergistic enhancement of the IRI activity of QAE appears to occur only when HaloTag – rather than any of the other tested tags – is attached N-terminally to QAE, and we did not find a similar effect for other IBPs. We also assessed the TH activity of HaloTag itself and found that the protein lacks this type of ice-binding activity.

### Ice-crystal shaping by HaloTag and fusion constructs

**Fig. 6:**
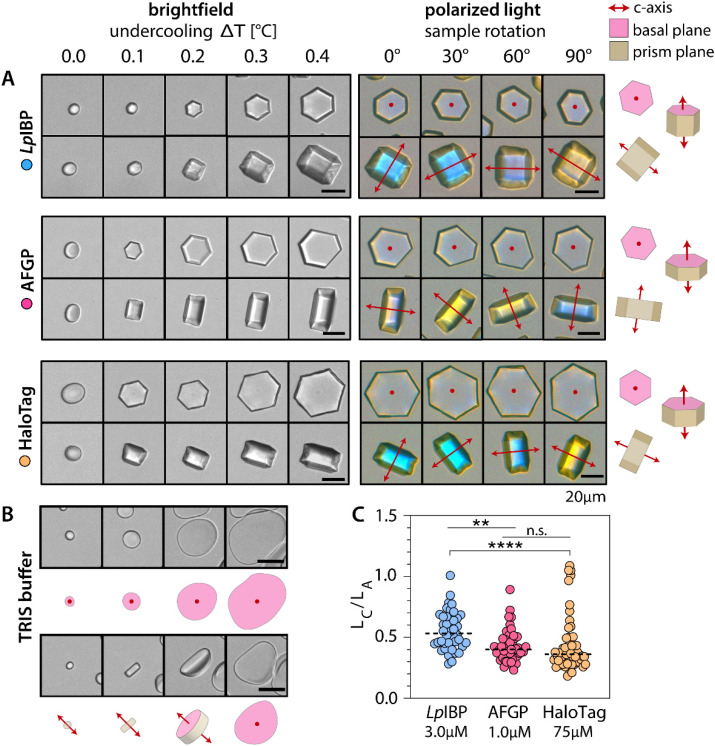
Ice crystal shaping patterns induced by HaloTag, *Lp*IBP and AFGP. (A) Left: Bright field images that follow single ice crystals with *Lp*IBP (3.0 μM) or AFGP (1.0 μM) or HaloTag (75 μM) in buffer (20 mM TRIS, 30% sucrose) upon cooling with 0.2 °C per minute. Right: Polarized optical microscopy images of the crystals on the left side at constant temperature (0.4 °C of undercooling), upon rotation of the microscope stage. All images taken with crossed polarizers and a first order retardation plate in place. Red dots indicate that the *c*-axis (crystal optical axis) is perpendicular to the viewing plane: then crystals have the same color as the background for all rotation angles. Red arrows indicate that the direction of the *c*-axis is parallel to the viewing plane: then the yellow or blue color indicates the crystal orientation with respect to the fixed orientation of the retardation plate. (B) Bright field images that follow single ice crystals in buffer (20 mM TRIS, 30% sucrose) without ice-binding protein upon cooling with 0.2 °C per minute. (C) *c*-Axis/*a*-axis length ratios (*L*_*c*_/*L*_*a*_) of multiple individual ice-crystals with protein at 0.4 °C of undercooling (*n* = 40–50). Dotted horizontal lines indicate the median *L*_*c*_/*L*_*a*_ values. Significant differences between groups are indicated by ** (*p* = 0.0013) and **** (*p* < 0.0001). All scale bars: 20 μm.

Next, we used polarized light microscopy to evaluate whether HaloTag binds ice. At sufficiently high concentrations, ice-binding proteins generate ice crystal morphologies upon cooling that are distinctly different from the ice crystal morphology observed in the absence of the IBPs. The formation of particular ice crystal facets indicates that the proteins likely interact with these planes. The use of polarized light microscopy allows us to determine the orientation of the ice crystal.^27^ Our considerations for choosing this method are in the SI (pages 3–7). Aiming to elucidate whether HaloTag binds to ice crystals and whether it has a preference for specific ice crystal planes (like most IBPs), we set out to monitor both the morphology and orientation of ice crystals undergoing growth upon cooling in the presence of the putative ice-binder HaloTag. We compared the results to those for two positive controls, the IBPs *Lp*IBP and AFGP (a synthetic analogue of AFGP8^27–29^), and one negative control, a sucrose solution without any proteins (Fig. 6). For all three proteins, the used concentration is equal to its IRRINA assay endpoint.

In the negative control, with only the buffer, ice crystals expand rapidly along the *a*-axis to obtain a rounded flat morphology (Fig. 6B). The rounded edges minimize the interfacial energy between the crystal surface and the surrounding solution. The flat morphology is in agreement with modeling studies that have shown that ice growth in the directions of the prism planes (*a*-axes) is considerably faster than in the direction of the basal plane (*c*-axis).^41^ In the presence of IBPs, the slowest growing ice crystal plane may not always be the same as the IBP binding plane, due to the different ice crystal growth rates along the *c*- and *a*-axis. *Lp*IBP has previously been established to have affinity for the basal plane {001} and the primary prism plane {100}.^22^ AFGP also binds to the primary prism plane, but it does not bind to the basal plane like *Lp*IBP.^27,42–44^ Both *Lp*IBP and AFGP induce hexagonal prismatic morphologies (Fig. 6A), which are in line with their ice-plane affinities. The *c*-axis and *a*-axis lengths (*L*_*c*_ and *L*_*a*_, respectively) of multiple individual crystals grown at 0.4 °C of undercooling were measured: *Lp*IBP (3.0 μM) gives an average *L*_*c*_/*L*_*a*_ = 0.55 ± 0.15, and AFGP (1.0 μM) gives an average *L*_*c*_/*L*_*a*_ = 0.43 ± 0.13. Both of these proteins slow down the rapid growth along the prism planes (*a*-axes), but that growth is still faster than growth along the basal planes (*c*-axis) (*L*_*c*_/*L*_*a*_ < 1). However, the *L*_*c*_/*L*_*a*_ for AFGP is significantly smaller than for *Lp*IBP, which could be caused by a weaker binding affinity for the primary prism plane.

Ice crystals grown in the presence of HaloTag (75 μM) have hexagonal shapes when viewed from along the *c*-axis, but appear rectangular when viewed from directions perpendicular to the *c*-axis (Fig. 6A). This marked shaping by HaloTag indicates that the protein tag interacts with ice crystal surfaces. We observe that most ice crystals grow faster in the direction of the *a*-axes than in the direction of the *c*-axis, with *L*_*c*_/*L*_*a*_ = 0.43 ± 0.22 at 0.4 °C of undercooling. This morphology resembles ice crystal growth in the presence of AFGP and suggests that HaloTag has a weak affinity for the primary prism plane of ice.

**Fig. 7:**
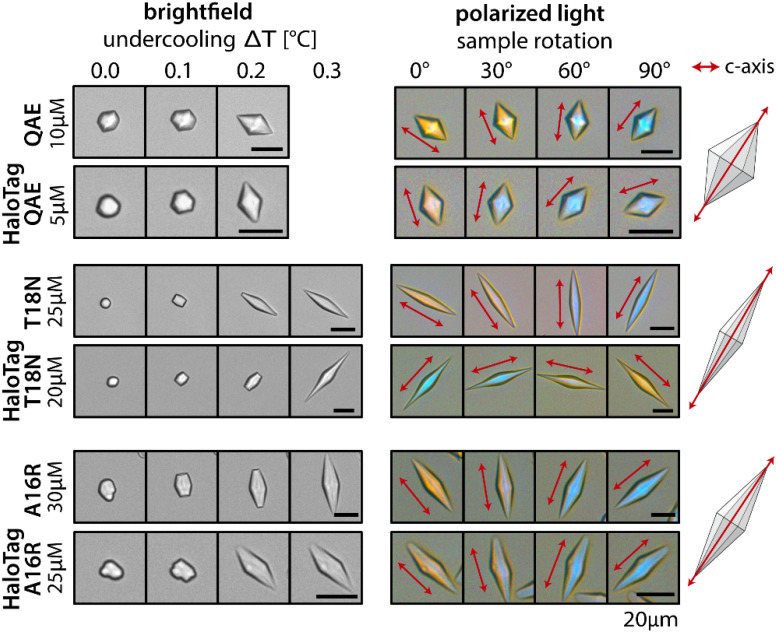
Ice crystal shaping patterns induced by untagged QAE and fusion constructs with the HaloTag and QAE. (Left) Bright field images that follow single ice crystals upon cooling with 0.2 °C per minute. (Right) Polarized optical microscopy images of the crystals on the left side at constant temperature, upon rotation of the microscope stage. All images taken with crossed polarizers and a first order retardation plate in place. The red dots indicate that the *c*-axis (optical axis of the crystal) is perpendicular to the viewing plane: then crystal has the same color as the background for all rotation angles. The red arrows indicate that the direction of the *c*-axis is parallel to the viewing plane: then the yellow or blue color indicates the crystal orientation with respect to the fixed orientation of the retardation plate. All scale bars: 20 μm.

Next, we evaluated if the ice crystal shaping activity of IBPs is altered by fusion to HaloTag using polarized optical microscopy. We imaged ice crystal shaping in the presence of QAE and its two mutants (T18N and A16R) with and without N-terminally attached HaloTag, because HaloTag enhances IRI activity in these cases. We found bipyramidal ice crystal shaping for QAE and its mutants, corresponding to previous studies on QAE and mutants without tag.^45^ The ice crystal shapes generated by QAE and the mutants T18N and A16R were found to be insensitive to the N-terminal attachment of HaloTag, although shaping activity is observed at slightly lower concentrations with attached HaloTag (Fig. 7).

## Discussion

Since protein tags are often employed to gain insight into the IBP structure and activity relations with fluorescence microscopy, it is essential to know whether such tags influence IBP activity. Previously it has already been reported that TH activity can be affected by fusion partners, and here we focused on the effect of protein tags on IRI activity.

Our results indicate that IRI activity of IBPs can be enhanced by fusion to a protein tag, but this enhancement appears unrelated to the size of the tag. Since the N-terminal SNAP-tag, the N-terminal mEos3.2, and C-terminal HaloTag do not influence IRI activity, these constructs are good candidates to investigate how the structures of IBPs without tag relate to their ice-binding activity by imaging the interaction with the ice crystal surface using fluorescence microscopy.

**Fig. 8:**
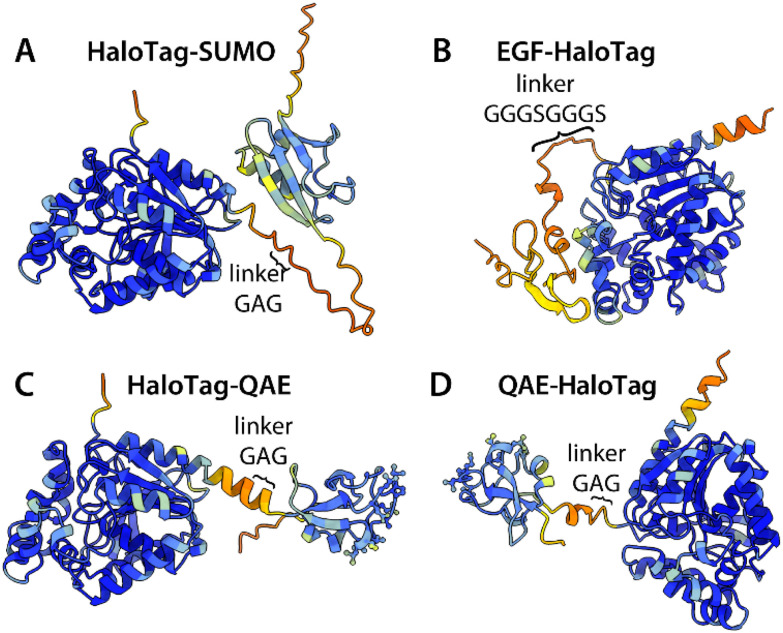
AlphaFold models of fusion constructs with HaloTag, indicating orientation and linker length. (A and B) HaloTag with SUMO at the C-terminus or EGF at the N-terminus. (C and D) HaloTag with QAE at the C- or N-terminus.

On the other hand, we identified a remarkable synergistic enhancement of IRI activity when HaloTag was N-terminally fused to QAE. Similarly, the IRI activities of two point mutants, T18N (with a mutation on the primary prism plane binding site), and A16R (with a mutation on the pyramidal plane binding site), are also increased upon N-terminal attachment of HaloTag. The IRI activities of QAE, T18N and A16R are increased by one order of magnitude. This suggests that the synergy is not related to binding to only one of the two preferred crystal planes of QAE. Most likely, the synergistic effect is related to the ice-binding activity of HaloTag itself: HaloTag shows moderate IRI activity, which was found to be inhibited when another protein is attached to one of the termini in two cases. We expect that the presumed IBS of HaloTag could be blocked by the specific non-ice-binding proteins used here, as suggested by the structural models of the fusion constructs (Fig. 8A and B). In addition to IRI activity, HaloTag induces facet formation on ice crystals which indicates that HaloTag interacts with ice. Our visual inspection of the crystal structure of HaloTag did not reveal a clear IBS. In future work, ice-biasing simulations^46^ could be performed to identify the IBS and crystal plane with which HaloTag interacts. Here we present ice-binding by HaloTag mainly as a potential explanation for the enhancement of IRI activity in the fusion construct HaloTag-QAE.

The synergistic enhancement in IRI activity was observed only when QAE and HaloTag are attached in the proper orientation: there is no synergy when QAE and HaloTag are simply mixed together, when HaloTag is attached at the C-terminus of QAE, or when HaloTag is fused to the N-terminus of wfAFP or *Lp*IBP. We hypothesize that the emergence of synergy relies on proper orientation of the two fusion partners: their IBS should be oriented in such a way that both fusion partners can interact with the ice simultaneously. In relation to this, also the linker sequence between the protein tag and the IBP can influence the conformation of the two proteins, and may be important for the presence or absence of synergy in IRI. Structural models of fusion constructs with QAE and protein tags indicate that the ice-binding site of QAE points away from the protein tag (Fig. 8C and D). Therefore we assume that fusion to the protein tag with the GAG linker does not cause steric hindrance in the interaction of QAE with ice. To investigate the effect of the linker length on IRI, we recommend to investigate IRI activity of HaloTag-QAE constructs with longer linkers in future research.

We hypothesize that HaloTag and QAE have to be fused together for strong synergy to occur, because HaloTag has a weaker ice-binding capacity than QAE. As QAE in the fusion construct binds to ice, HaloTag will also be near to the ice and may be able to interact with ice more quickly than separate HaloTag. Here we have only investigated the addition separate HaloTag and QAE in an equimolar ratio to compare with the fusion construct, but to investigate the synergistic effect further, other concentration ratios could be used in future research.

Interestingly, we note that the attachment of different protein tags to QAE influences IRI and TH in different ways. HaloTag-QAE shows strongly enhanced IRI activity compared to QAE, while its TH activity increases only moderately. Fusion to mEos3.2 does not influence the IRI activity of QAE, but increases TH more strongly than fusion to HaloTag. This suggests that IRI and TH activity have different ice-growth inhibition mechanisms. Molecular simulations have indicated that TH activity is influenced by the ability of the protein to resist engulfment by ice upon cooling, which depends on the size and shape of the non-ice-binding site (NBS).^11^ Fusion of IBPs to a protein tag enlarges the size of the NBS and alters its shape, which may clarify why protein tags moderately increase TH activity. IRI activity of fusion constructs may depend less on the NBS and more on interactions between the IBS and ice. We hypothesize that enlargement of the IBS may lead to enhancement of IRI activity. This could be the reason that HaloTag is able to enhance IRI upon fusion to QAE (assuming that HaloTag indeed interacts with ice), while mEos3.2 does not alter IRI activity. Protein engineering has shown that TH activity can also be increased by enlarging the size of the IBS, but this effect is case-specific.^12,47^ We hypothesize that in case of HaloTag-QAE, the enlargement of the ice-binding surface does not significantly contribute to increase in TH activity, given that HaloTag itself is not TH active. In IRI conditions, reversible interactions with ice are sufficient to inhibit ice-growth.^9^ HaloTag itself may have a very short interaction time with the ice, which is sufficient for IRI but not TH. In fusion constructs with IBPs, HaloTag may strongly enhance IRI through interaction with ice, but not TH. We recommend the performance of MD simulations^11,48^ on fusion constructs with QAE and various protein tags to test these hypotheses. In that way, mechanisms for ice-growth inhibition in TH and IRI may be elucidated, offering perspective on how to enhance these types of ice-binding activity.

## Conclusions

In this work, we have addressed how the ice-binding activities of IBPs are influenced upon fusion to protein tags that facilitate visualization of IBPs on ice-crystal surfaces using fluorescence microscopy. We found that in most cases, IRI activity of IBPs is not affected by the attachment of a protein tag. Specifically, for QAE, we have shown that mEos3.2, SNAP-tag, and HaloTag (when attached C-terminally) do not affect its IRI activity. However, we also found that HaloTag itself has moderate IRI activity and strongly enhances the IRI activity of QAE when attached N-terminally. The hexagonal prismatic ice-crystal shaping induced by HaloTag points to a weak affinity for the primary prism plane, but it does not influence the shaping induced by QAE upon N-terminal attachment. Even though a putative IBS of the HaloTag, and putative molecular mechanism for the synergistic effect in IRI remain elusive for now, our results do point to the possibility for strong synergistic enhancement of IRI activities of IBPs *via* judiciously chosen fusion partners with moderate ice-binding activity. Conveniently, the tags that do not affect IBP activity can be employed to study structure–activity relations of IBPs using fluorescence microscopy. Here we applied polarized light microscopy to determine the orientation of individual crystals and quantify growth along their *c*- and *a*-axes. In the future, we envision performing both polarized light microscopy and fluorescence microscopy on the same sample. This would enable us to identify the ice-plane affinity of novel compounds in smaller sample volumes and higher throughput than presently possible. It would also allow for the first plane-resolved super-resolution microscopy experiments on the dynamics of ice-binders on ice.

## Author contributions

DvdB: conceptualization, resources, investigation, formal analysis, visualization, writing – original draft. SNG: investigation, formal analysis, writing – review and editing. TPH: supervision, resources. RdV: writing – review and editing. IKV: funding acquisition, conceptualization, supervision, writing – review and editing.

## Conflicts of interest

The authors declare no conflict of interest.

## Supplementary Material

NR-018-D5NR04805B-s001

## Data Availability

Data for this article, including IRI and ice-crystal shaping data, are available at 4TU.ResearchData repository. See DOI: https://doi.org/10.4121/7f576fbd-6a14-4f7a-aeea-6374dec811a4. Supplementary information (SI) is available, including TH methods and data, extended methods for ice-crystal shaping assay, protein purification and peptide synthesis, all protein sequences and AlphaFold models of fusion constructs. See DOI: https://doi.org/10.1039/d5nr04805b.

## References

[cit1] Bar Dolev M. , Braslavsky I. and Davies P. L., in Annual Review of Biochemistry, ed. R. D. Kornberg, 2016, vol. 85, pp. 515–542 10.1146/annurev-biochem-060815-014546 27145844

[cit2] Voets I. K. (2017). Soft Matter.

[cit3] Tas R. P., Sampaio-Pinto V., Wennekes T., Laake L. W., Voets I. K. (2021). EMBO Rep..

[cit4] Duman J. G., Wisniewski M. J. (2014). Environ. Exp. Bot..

[cit5] Pham Q. T. and Mawson R. F., in Quality in Frozen Foods, ed. M. C. Erickson and Y.-C. Hung, Springer US, Boston, MA, 1997, pp. 67–91

[cit6] Murray K. A., Gibson M. I. (2022). Nat. Rev. Chem..

[cit7] Monalisa K., Shibata M., Hagiwara T. (2023). Food Hydrocolloids.

[cit8] Mitchell D. E., Fayter A. E. R., Deller R. C., Hasan M., Gutierrez-Marcos J., Gibson M. I. (2019). Mater. Horiz..

[cit9] Tas R. P., Hendrix M. M. R. M., Voets I. K. (2023). Proc. Natl. Acad. Sci. U. S. A..

[cit10] Chasnitsky M., Braslavsky I. (2019). Philos. Trans. R. Soc., A.

[cit11] Thosar A. U., Cai Y., Marks S. M., Vicars Z., Choi J., Pallath A., Patel A. J. (2024). Proc. Natl. Acad. Sci. U. S. A..

[cit12] Baardsnes J., Kuiper M. J., Davies P. L. (2003). J. Biol. Chem..

[cit13] Wang C., Oliver E. E., Christner B. C., Luo B.-H. (2016). Biochemistry.

[cit14] Wang C., Pakhomova S., Newcomer M. E., Christner B. C., Luo B.-H. (2017). PLoS One.

[cit15] Berger T., Meister K., DeVries A. L., Eves R., Davies P. L., Drori R. (2019). J. Am. Chem. Soc..

[cit16] Gaukel V., Leiter A., Spieß W. E. L. (2014). J. Food Eng..

[cit17] Monalisa K., Shibata M., Hagiwara T. (2021). Food Biophys..

[cit18] Los G. V., Encell L. P., McDougall M. G., Hartzell D. D., Karassina N., Zimprich C., Wood M. G., Learish R., Ohana R. F., Urh M., Simpson D., Mendez J., Zimmerman K., Otto P., Vidugiris G., Zhu J., Darzins A., Klaubert D. H., Bulleit R. F., Wood K. V. (2008). ACS Chem. Biol..

[cit19] Wilhelm J., Kühn S., Tarnawski M., Gotthard G., Tünnermann J., Tänzer T., Karpenko J., Mertes N., Xue L., Uhrig U., Reinstein J., Hiblot J., Johnsson K. (2021). Biochemistry.

[cit20] Middleton A. J., Brown A. M., Davies P. L., Walker V. K. (2009). FEBS Lett..

[cit21] Lauersen K. J., Brown A., Middleton A., Davies P. L., Walker V. K. (2011). Cryobiology.

[cit22] Middleton A. J., Marshall C. B., Faucher F., Bar-Dolev M., Braslavsky I., Campbell R. L., Walker V. K., Davies P. L. (2012). J. Mol. Biol..

[cit23] Sicheri F., Yang D. S. C. (1995). Nature.

[cit24] Knight C. A., Cheng C. C., DeVries A. L. (1991). Biophys. J..

[cit25] Zhang M., Chang H., Zhang Y., Yu J., Wu L., Ji W., Chen J., Liu B., Lu J., Liu Y., Zhang J., Xu P., Xu T. (2012). Nat. Methods.

[cit26] Mollwitz B., Brunk E., Schmitt S., Pojer F., Bannwarth M., Schiltz M., Rothlisberger U., Johnsson K. (2012). Biochemistry.

[cit27] Budke C., Dreyer A., Jaeger J., Gimpel K., Berkemeier T., Bonin A. S., Nagel L., Plattner C., Devries A. L., Sewald N., Koop T. (2014). Cryst. Growth Des..

[cit28] Peltier R., Evans C. W., DeVries A. L., Brimble M. A., Dingley A. J., Williams D. E. (2010). Cryst. Growth Des..

[cit29] Urbańczyk M., Góra J., Latajka R., Sewald N. (2017). Amino Acids.

[cit30] Budke C., Heggemann C., Koch M., Sewald N., Koop T. (2009). J. Phys. Chem. B.

[cit31] Abramson J., Adler J., Dunger J., Evans R., Green T., Pritzel A., Ronneberger O., Willmore L., Ballard A. J., Bambrick J., Bodenstein S. W., Evans D. A., Hung C.-C., O'Neill M., Reiman D., Tunyasuvunakool K., Wu Z., Žemgulytė A., Arvaniti E., Beattie C., Bertolli O., Bridgland A., Cherepanov A., Congreve M., Cowen-Rivers A. I., Cowie A., Figurnov M., Fuchs F. B., Gladman H., Jain R., Khan Y. A., Low C. M. R., Perlin K., Potapenko A., Savy P., Singh S., Stecula A., Thillaisundaram A., Tong C., Yakneen S., Zhong E. D., Zielinski M., Žídek A., Bapst V., Kohli P., Jaderberg M., Hassabis D., Jumper J. M. (2024). Nature.

[cit32] Chao H., DeLuca C. I., Davies P. L., Sykes B. D., Sönnichsen F. D. (1994). Protein Sci..

[cit33] Antson A. A., Smith D. J., Roper D. I., Lewis S., Caves L. S. D., Verma C. S., Buckley S. L., Lillford P. J., Hubbard R. E. (2001). J.
Mol. Biol..

[cit34] Garnham C. P., Natarajan A., Middleton A. J., Kuiper M. J., Braslavsky I., Davies P. L. (2010). Biochemistry.

[cit35] DeLuca C. I., Davies P. L., Ye Q., Jia Z. (1998). J. Mol. Biol..

[cit36] Graether S. P., DeLuca C. I., Baardsnes J., Hill G. A., Davies P. L., Jia Z. (1999). J. Biol. Chem..

[cit37] Olijve L. L. C., Oude Vrielink A. S., Voets I. K. (2016). Cryst. Growth Des..

[cit38] Huang H.-W., Mohan S. K., Yu C. (2010). Biochem. Biophys. Res. Commun..

[cit39] Sheng W., Liao X. (2002). Protein Sci..

[cit40] Guerrero F., Ciragan A., Iwaï H. (2015). Protein Expression Purif..

[cit41] Rozmanov D., Kusalik P. G. (2012). J. Chem. Phys..

[cit42] Knight C. A., Driggers E., DeVries A. L. (1993). Biophys. J..

[cit43] Tsuda S., Yamauchi A., Uddin Khan N. M. M., Arai T., Mahatabuddin S., Miura A., Kondo H. (2020). Biomolecules.

[cit44] Meister K., DeVries A. L., Bakker H. J., Drori R. (2018). J. Am. Chem. Soc..

[cit45] Deluca C. I. , Chao H., Sonnichsen F. D., Sykes B. D. and Davies P. L., Effect of Type III Antifreeze Protein Dilution and Mutation on the Growth Inhibition of Ice, 1996, vol. 71 10.1016/S0006-3495(96)79476-6 PMC1233724 8913575

[cit46] Naullage P. M., Metya A. K., Molinero V. (2020). J. Chem. Phys..

[cit47] Scholl C. L., Davies P. L. (2023). FEBS Lett..

[cit48] Gerhäuser J., Gaukel V. (2025). Langmuir.

