# Supplementary material for: Activity of ice-binding proteins can be markedly enhanced by protein tags
Source: Nanoscale. 2026 Mar 11;18(14):7767–77. doi: 10.1039/d5nr04805b (PMC12978126; doi:10.1039/d5nr04805b)
Supplement: NR-018-D5NR04805B-s001 [file NR-018-D5NR04805B-s001.pdf]

Supplementary Information (SI) for Nanoscale.
This journal is © The Royal Society of Chemistry 2026

Supporting Information:

Activity of ice-binding proteins can be markedly enhanced by protein tags

Daniëlle van den Broek,[a] Sanne N. Giezen, [a] Tim P. Hogervorst,[a] Renko de Vries[b] and Ilja K. Voets [a,1]

[a] *Laboratory of Self Organizing Soft Matter, Department of Chemical Engineering and Chemistry and Institute for Complex Molecular Systems, Eindhoven University of Technology, Eindhoven, The Netherlands*

[b] *Department of Physical Chemistry and Soft Matter, Wageningen University and Research, Wageningen, The Netherlands.*

[1] *Corresponding author*

Table of contents

1. **TH activities** **2**

Methods: Thermal hysteresis (TH) assay 2

Figure S1: TH activities of untagged QAE, mEos3.2-QAE, HaloTag-QAE and QAE-HaloTag 2

2. **Ice-crystal shaping with polarized light microscopy** **3**

Theory and methods: Using birefringence in ice-crystals to determine ice-crystal orientation with polarized light microscopy 3

Figure S2: Overview of the polarized optical microscopy set-up 4

Figure S3: Illustration of birefringence by ice-crystals with multiple orientations in polarized light microscopy 5

Figure S4: Illustration of birefringence from ice-crystals in multiple orientations in polarized light microscopy with the addition of a first order retardation plate 6

Methods: Ice crystal shaping sample preparation with sucrose 7

3. **Protein constructs** **7**

Figure S5: AlphaFold models of the fusion constructs with HaloTag 7

Table S1: Protein sequences
11 **Table S2:** Protein data bank (PDB) codes
13
Methods: Protein expression and purification of HaloTag constructs
13
Figure S6: SDS-PAGE gels
14
Figure S7: QToF mass spectrometry
20

4. **Solid phase peptide synthesis**
21
Methods: Synthesis of AFGP analogue and wfAFP
21

5. **References**
24

1. TH activities

Methods: Thermal hysteresis (TH) assay

TH activities were measured using an in-house built nanoliter osmometer with 0.001°C temperature accuracy, based on a previously described set-up (1,2). In short, a metal disk with six holes of 0.6 mm was placed onto the cooling plate of the stage. Type B Cargille Immersion oil was inserted into these holes, followed by a tiny droplet of the protein solution of interest with a concentration of 140µM in buffer (20 mM TRIS, pH=7.5).

The samples were imaged using an Olympus BX53M optical microscope, equipped with an Olympus LMPLFLN 50x (NA 0.50) objective and a Lumenera Infinity1 camera (resolution approximately 0.1µm per pixel). The droplets were quickly frozen by cooling rapidly to -35°C and melted back until a single crystal was observed within a droplet. The melting temperature was determined as the temperature at which this single crystal kept shrinking in size. The crystal was equilibrated at 0.05°C below the melting temperature for two minutes, followed by cooling with a rate of 0.1°C/min. During the cooling ramp, snapshots were captured every 0.5 seconds. The freezing temperature was determined as the temperature at which rapid burst growth of the crystal was observed, upon which the whole droplet was eventually frozen. Then the TH activity was calculated as the difference between the melting temperature and the freezing temperature.

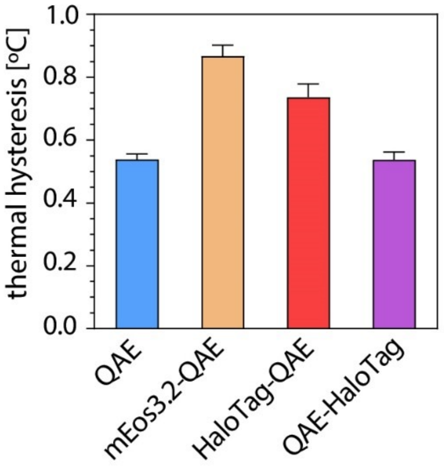

Figure S1. Thermal hysteresis (TH) activity of tagged and untagged QAE. Thermal hysteresis gaps for single ice-crystals at protein concentrations of 140µM, with at least four measurements per protein.

2. Ice-crystal shaping with polarized optical microscopy

Theory and methods: Using birefringence in ice-crystals to determine ice-crystal orientation with polarized light microscopy

To date, preferential binding of IBPs to certain ice-crystal planes has often been studied using the ice-etching (3) or fluorescence based ice-plane affinity (FIPA) method (4). With both techniques, IBPs in solution are allowed to bind to a hemispheric, macroscopic ice crystal with known orientation. In ice-etching, rough patches on the crystal indicate binding of the IBP, while in FIPA a fluorescent tag on the IBP is used to facilitate detection. Due to the need for macroscopic ice crystals with known orientation, the FIPA method is laborious. Imaging morphologies of microscopic crystals requires less protein and may suggest a particular ice crystal plane specificity, especially when fluorescence microscopy is used to visualize accumulation of IBPs on crystal surfaces (5–15), but this method does not allow for precise determination of the ice crystal orientation.

Polarized light microscopy can be used to determine the orientation of microscopic crystals growing in the presence of IBPs, as we have shown in this work. This technique rests on the anisotropic nature of the hexagonal ice crystal lattice: the spatial distribution of atoms within the lattice is non-uniform along all directions. Because of this the optical properties of ice depend on the crystal orientation with respect to the incoming light. Ice crystals have a single optical axis, which is also referred to as the c-axis (16). When light enters the crystal along the optical axis, it will pass through at a single velocity. However, when light enters along any other axis, it will be split into two rays which have a mutually perpendicular orientation and different refractive indices. One of the rays is called the ordinary ray, which has the same velocity along all directions of the crystal. The other ray is the extraordinary ray, which travels through the crystal with a velocity that depends on the crystal orientation (17).

The phenomenon of double refraction of light is called birefringence. Birefringence is defined as the difference between the refractive indices of the extraordinary and ordinary ray (equation 1).

$$\Delta n(\lambda) = n_e(\lambda) - n_o(\lambda) \quad (1)$$

$$n_e(\lambda) = \frac{n_o(\lambda)}{\sqrt{1 + \left(\frac{n_o(\lambda)^2}{n_\varepsilon(\lambda)^2} - 1\right) sin^2\theta}} \quad (2)$$

$$n_o(\lambda) = n_\omega(\lambda) \quad (3)$$

n_ε and n_ω correspond to the refractive indices of the extraordinary and ordinary ray. Both parameters are dependent on the wavelength of the incoming light. $n_e$and n_o refer to the refractive indices that are projected along the plane that is perpendicular to the incident light. This shows how the refractive index of the extraordinary ray depends on the angle θ between the optical axis of the crystal and the incoming light (equation 2), while the refraction of the ordinary ray is independent of the angle (equation 3). When θ=0°, birefringence does not occur because the incoming light is parallel to the c-axis (optical axis) of the crystal. The level of birefringence is at its maximum when the c-axis is perpendicular to the incident light: θ=90°. The measured refraction indices of the ordinary and extraordinary rays in ice crystals indicate that the birefringence $\Delta n(\lambda)$ in ice crystals is ± 0.0015 (17).

We apply polarized optical microscopy to determine the orientation of birefringent ice crystals. This requires the sample to be in between two polarizing light filters with a crossed orientation (Figure S2). When the incoming light that vibrates in all directions passes through the first polarizer it will become plane polarized, vibrating in only one direction. Next the light passes through the crystal sample, which can cause birefringence. If the extraordinary ray travels more slowly through the crystal compared to the ordinary ray, the rays are out of phase when they exit the crystal. As mentioned above, the degree of birefringence is dependent

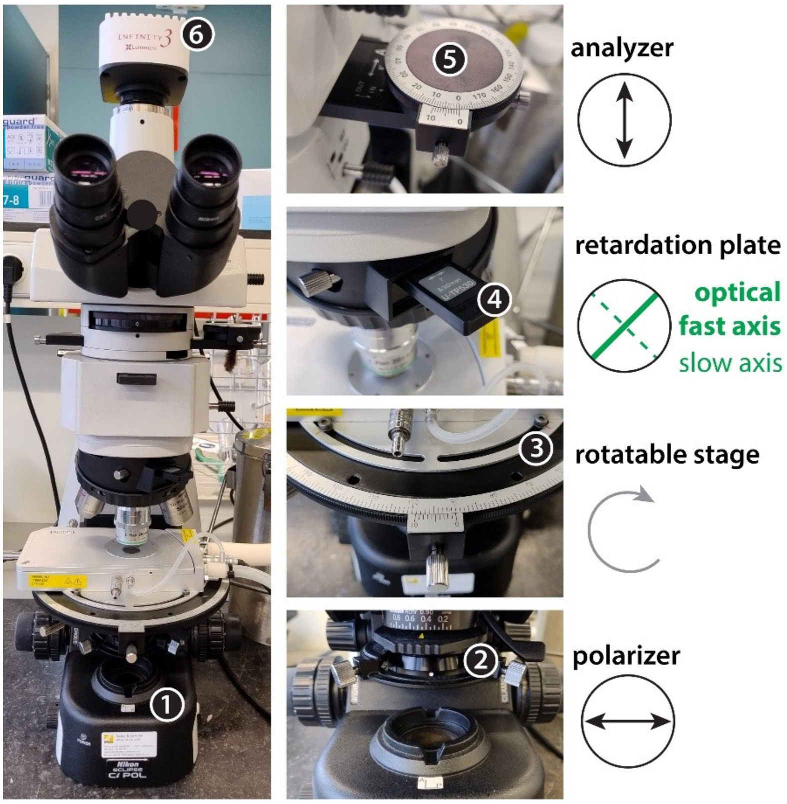

Figure S2. Overview of the polarized optical microscopy set-up. (1) light source, (2) polarizer, (3) rotatable stage, (4) first order (full wave) retardation plate (5) analyzer (second polarizer that is in crossed orientation with respect to the first polarizer), (6) color camera.

on the angle θ between the c-axis and the incident light. However, the magnitude of the phase difference between the two rays also depends on the azimuthal angle α between the c-axis and the polarizer. When $\alpha=0^o$ or $\alpha=90^o$, birefringence will occur but there will be no optical path difference between the two rays when they exit the crystal. The optical path difference is at its maximum for $\alpha= \pm 45^o$. When the two rays are out of phase they are combined again when they pass through the second polarizer, which is called the analyzer. The optical path difference between the rays results in a particular interference color of light (18), as depicted in the Michel-Levy chart or the Raith-Sørensen chart (19) (20). The magnitude of the optical path difference, also called retardation, depends on the degree of birefringence and the thickness of the crystal (equation 4).

$$retardation\ [nm] = \Delta n(\lambda) \times thickness\ [nm] \qquad (4)$$

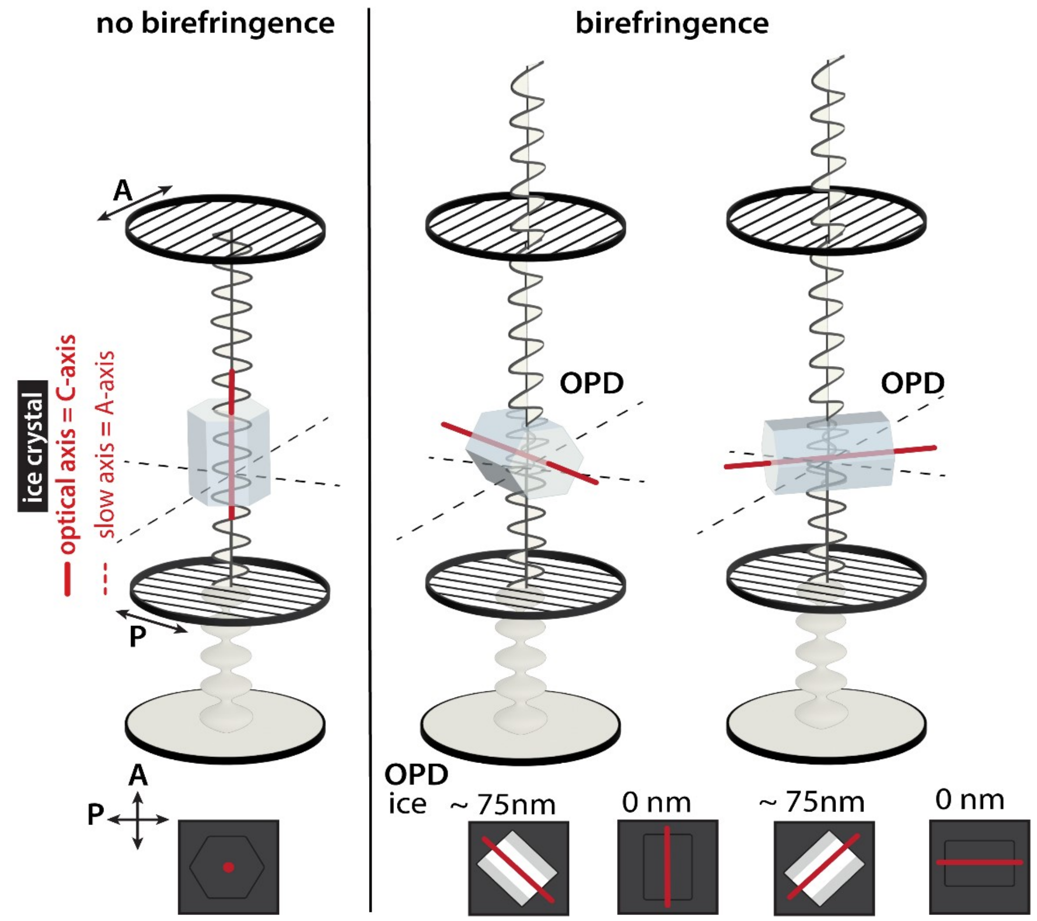

Figure S3. Illustration of birefringence by ice-crystals with multiple orientations in polarized light microscopy. (a) Birefringence does not occur when the c-axis is oriented parallel to the light path, so the crystal appears dark like the background. (b) Birefringence occurs when the c-axis is perpendicular to the light path. The ice crystal can induce an optical path difference (OPD) between the two light rays that are formed upon double refraction, corresponding to a white light signal. The magnitude depends on the azimuthal angle α between the c-axis and the polarizer.

The crystals with a thickness of 50µm - which we have in our experiments - would give a relatively small maximum retardation of ±75nm, which corresponds to a faint white light signal (Figure S3). To enhance the contrast when imaging such weakly birefringent crystals, we applied a first order retardation plate. It is inserted into the light path between the sample and the analyzer, with its optical and slow axes rotated 45° with respect to the polarizer and analyzer. The plate adds a retardation of 530nm to every wave that passes through it. Where no crystals are present the view becomes purple, because the blue and red light becomes elliptically polarized by the added retardation, while the green light remains plane polarized and is blocked by the analyzer (21). If an ice crystal is oriented with the c-axis perpendicular with the light path and α= ±45°, either the blue or the red light stays plane polarized while the other colors obtain elliptical polarization. Then an interference color becomes visible that indicates the orientation of the crystal. When and α= **+**45° (c-axis is aligned with the optical axis of the retardation plate), the retardation is additive and the crystal shows a blue color. When the crystal is rotated and α= **−**45° (c-axis is perpendicular to the optical axis of the retardation plate), the retardation is additive and the crystal shows a

pink color (Figure S4). To enhance the contrast of the images further, we apply white balance correction to the background before acquisition. Alternatively, the white balance correction can be applied afterwards with an automatic ImageJ macro (22).

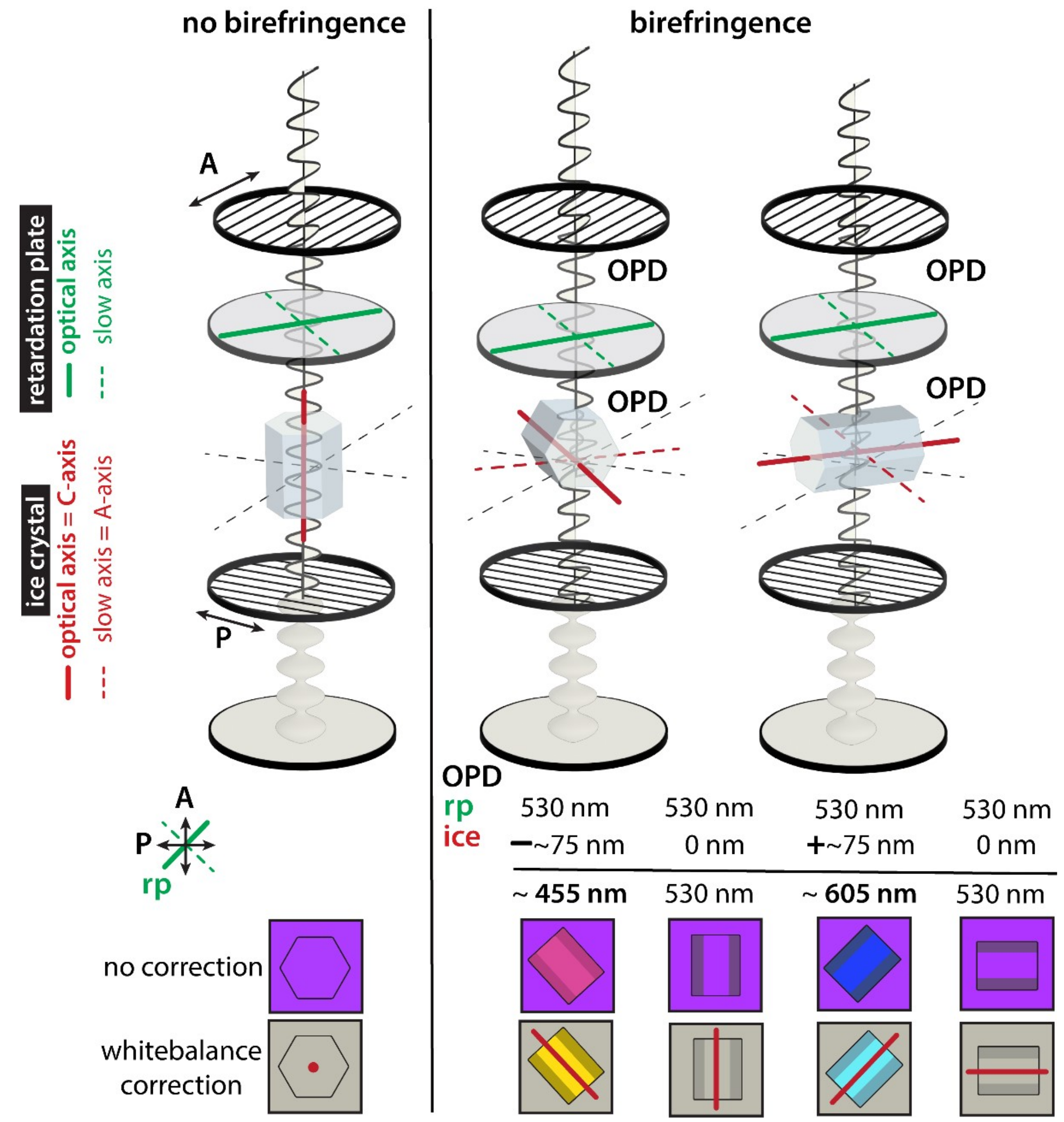

Figure S4. Illustration of birefringence from ice-crystals in multiple orientations in polarized light microscopy with the addition of a first order retardation plate. The retardation plate is a birefringent material that adds a optical path difference (OPD) of 530nm to every light wave that passes through it. This causes the background to become purple. The background appears grey after white balance correction, which is done to enhance contrast. (a) Birefringence does not occur when the c-axis is oriented parallel to the light path, so the crystal appears purple like the background. (b) Birefringence occurs when the c-axis is perpendicular to the light path. Depending on the azimuthal angle α between the c-axis and the polarizer, the OPD induced by the crystal is subtractive or additive to the OPD induced by the retardation plate.

Met

The IBP concentration during the shaping assay should be in the right window for observation of characteristic crystal facets. If the concentration is too low, the IBPs are not able to induce shaping. If it the concentration is too high, crystals remain too small to observe birefringence, after which a burst-like growth can occur. We have determined that this shaping assay can best be performed at the IRRINA assay endpoint (i.e. the lowest concentrations that show complete IRI activity): crystals grow gradually and obtain characteristic facets.

We supplement ice-crystal shaping samples with 30% sucrose to lower the ice-volume fraction and slow down the melting process, so that multiple individual ice-crystals can be observed using a Linkam cooling stage (precision ±0.1°C). To perform ice crystal shaping experiments without sucrose, a cooling stage with more precise temperature control would be required (±0.01°C). The amount of sucrose applied by us is also in line with previously published ice-crystal shaping experiments by Budke et al. (23)

We acknowledge that the ice-crystal shaping activity may be emphasized by the addition of sucrose. However, we point out that the negative control (30% sucrose without protein) does not induce ice-crystal shaping (Figure 6B). Furthermore, samples with non-ice-binding proteins in 30% sucrose do not induce ice-crystal shaping either (data not shown). The finding that HaloTag does give ice-crystal shaping is a qualitative indication that this protein does have interaction with the ice-crystal surface.

3. Protein constructs

Figure S5 Alphafold models of the fusion constructs with protein tags, IBPs and non-ice-binding proteins in this study, with indication of the used linker sequences.

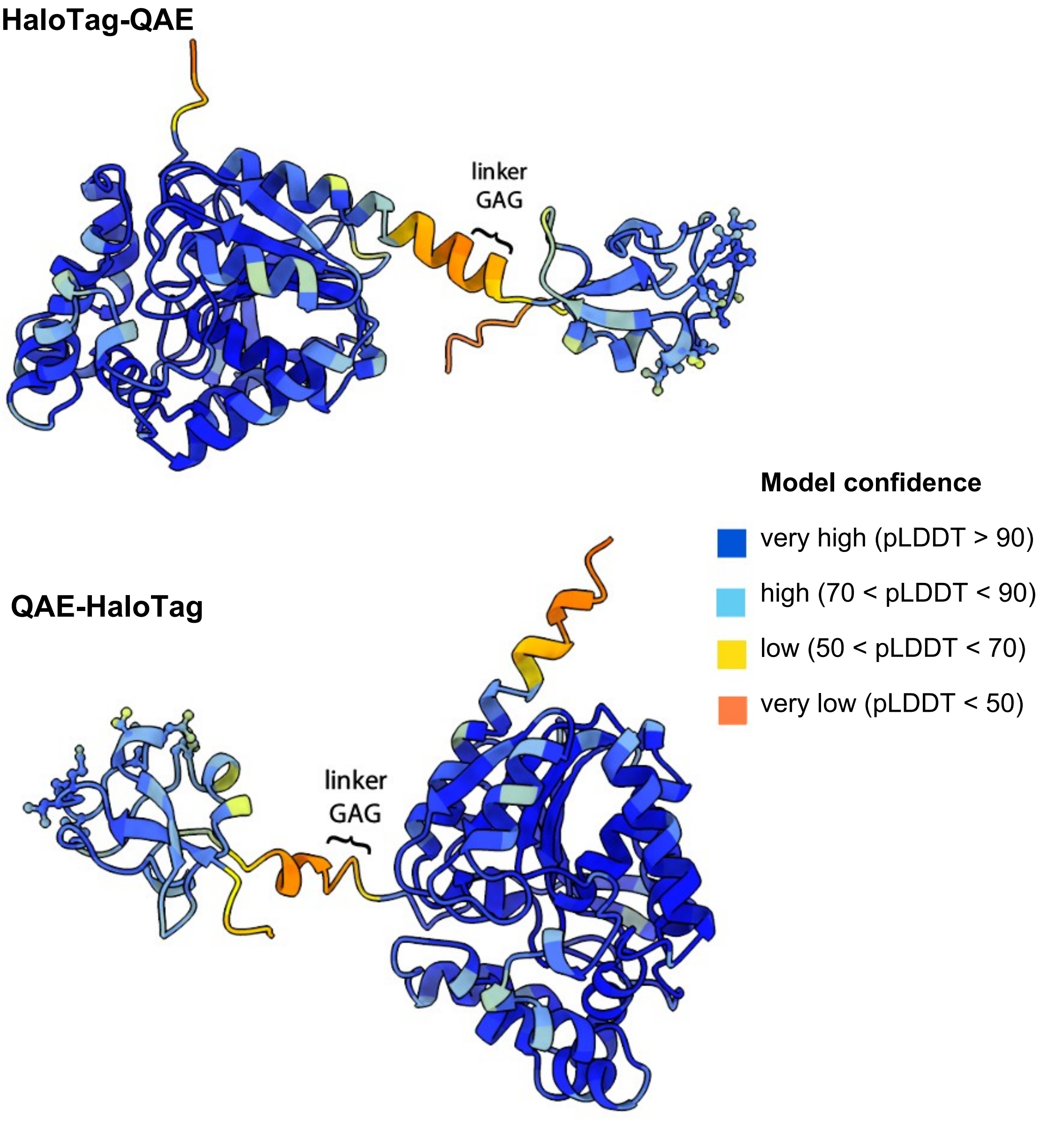

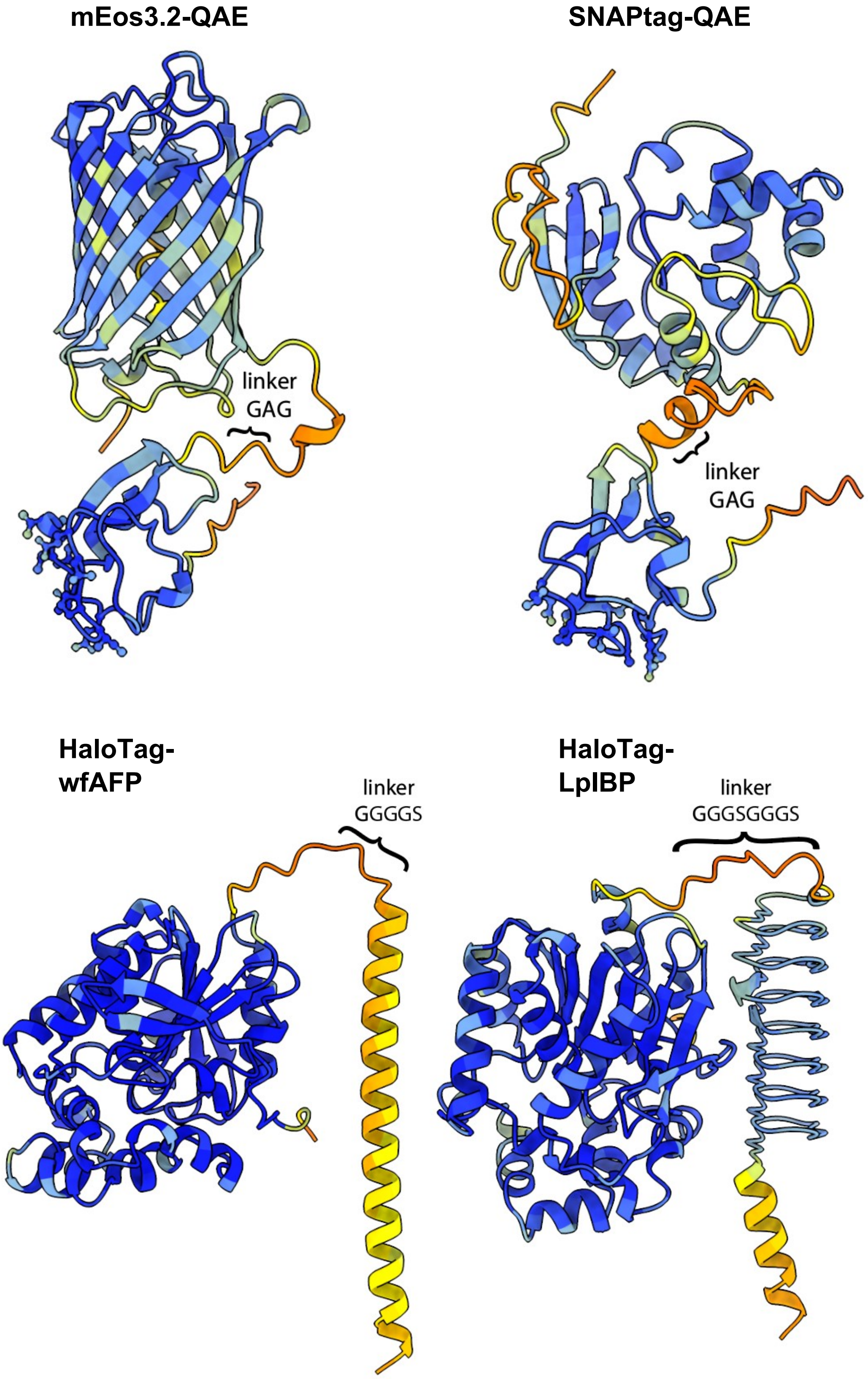
mEos3.2-QAE
linker
GAG
SNAPtag-QAE
linker
GAG
HaloTag-
wfAFP
linker
GGGGS
HaloTag-
LpIBP
linker
GGGSGGGS

HaloTag-SUMO

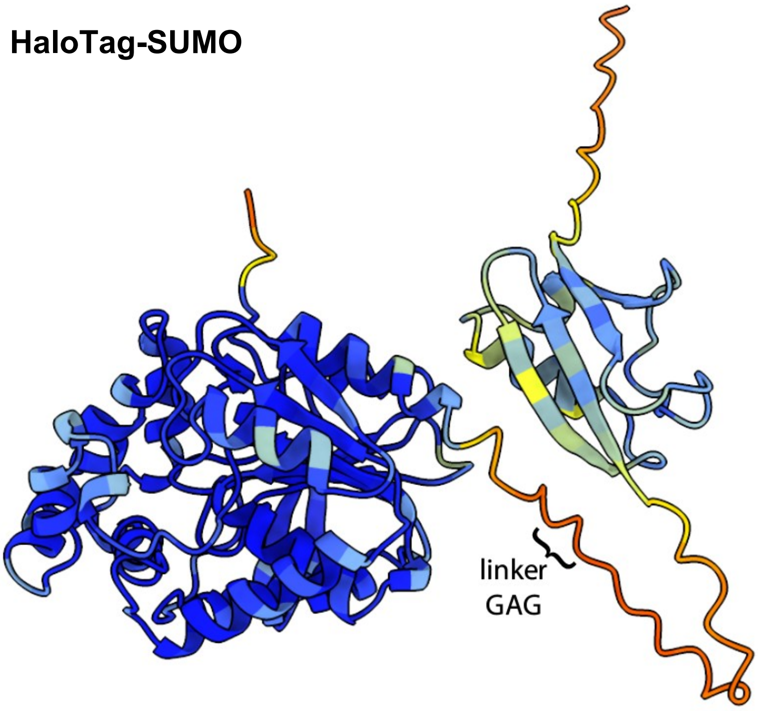

EGF-HaloTag

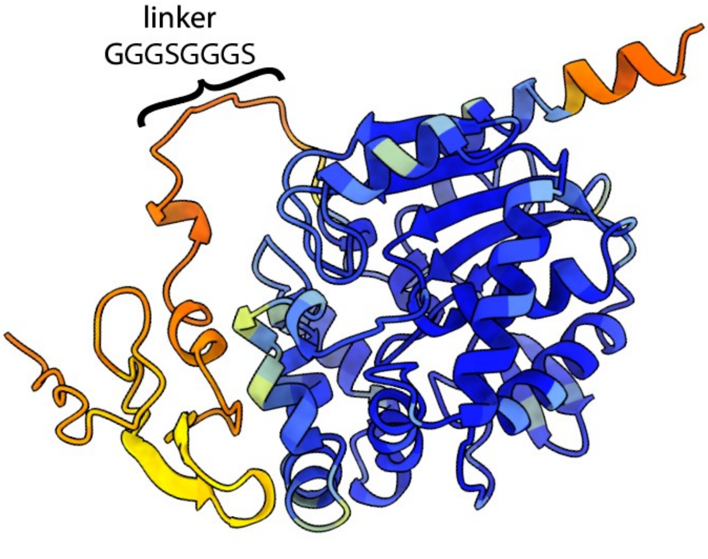

Table S1. Protein sequences

**QAE**-6xHIS	MGMNQASVVANQLIPINTALTLVMMRSEVVTPVGIPAEDIPRLVSMQVNRAVP<br>LGTTLMPDMVKGYAALEHHHHHH*
**HaloTag**-GAG-**QAE**-6xHIS	MGAEIGTGFPFDPHYVEVLGERMHYVDVGPRDGTPVLFLHGNPTSSYVWRNII<br>PHVAPTHRCIAPDLIGMGKSDKPDLGYFFDDHVRFMDAFIEALGLEEVVLVIH<br>DWGSALGFHWAKRNPERVKGIAFMEFIRPIPTWDEWPEFARETFQAFRTTDVG<br>RKLIIDQNVFIEGTLPMGVVRPLTEVEMDHYREPFLNPVDREPLWRFPNELPI<br>AGEPANIVALVEEYMDWLHQSPVPKLLFWGTPGVLIPPAEAARLAKSLPNCKA<br>VDIGPGLNLLQEDNPDLIGSEIARWLSTLEISGEFGAGMNQASVVANQLIPIN<br>TALTLVMMRSEVVTPVGIPAEDIPRLVSMQVNRAVPLGTTLMPDMVKGYAALE<br>HHHHHH*
**mEos3.2**-GAG-**QAE**-6xHIS	MGSAIKPDMKIKLRMEGNVNGHHFVIDGDGTGKPFEGKQSMDLEVKEGGPLPF<br>AFDILTTAFHYGNRVFAKYPDNIQDYFKQSFPKGYSWERSLTFEDGGICNARN<br>DITMEGDTFYNKVRFYGTNFPANGPVMQKKTLKWEPSTEKMYVRDGVLTGDIE<br>MALLLEGNAHYRCDFRTTYKAKEKGVKLPGAHFVDHCIEILSHDKDYNKVKLY<br>EHAVAHSGLPDNARREFGAGMNQASVVANQLIPINTALTLVMMRSEVVTPVGI<br>PAEDIPRLVSMQVNRAVPLGTTLMPDMVKGYAALEHHHHHH*
6xHIS-thrombin_site-**SNAPtag**-GAG-**QAE**-6xHIS	MGSSHHHHHHSSGLVPRGSHMMGDKDCEMKRTTLDSPLGKLELSGCEQGLHRI<br>IFLGKGTSAADAVEVPAPAAVLGGPEPLMQATAWLNAYFHQPEAIEEFPVPAL<br>HHPVFQQESFTRQVLWKLLKVVKFGEVISYSHLAALAGNPAATAAVKTALSGN<br>PVPILIPCHRVVQGDLDVGGYEGGLAVKEWLLAHEGHRLGKPGLGEFGAGMNQ<br>ASVVANQLIPINTALTLVMMRSEVVTPVGIPAEDIPRLVSMQVNRAVPLGTTL<br>MPDMVKGYAALEHHHHHH*
6xHIS-thrombin_site-**QAE**-GAG-**HaloTag**-6xHIS	MGSSHHHHHHSSGLVPRGSHMMGMNQASVVANQLIPINTALTLVMMRSEVVTP<br>VGIPAEDIPRLVSMQVNRAVPLGTTLMPDMVKGYAALEEFGAGAEIGTGFPFD<br>PHYVEVLGERMHYVDVGPRDGTPVLFLHGNPTSSYVWRNIIPHVAPTHRCIAP<br>DLIGMGKSDKPDLGYFFDDHVRFMDAFIEALGLEEVVLVIHDWGSALGFHWAK<br>RNPERVKGIAFMEFIRPIPTWDEWPEFARETFQAFRTTDVGRKLIIDQNVFIE<br>GTLPMGVVRPLTEVEMDHYREPFLNPVDREPLWRFPNELPIAGEPANIVALVE<br>EYMDWLHQSPVPKLLFWGTPGVLIPPAEAARLAKSLPNCKAVDIGPGLNLLQE<br>DNPDLIGSEIARWLSTLEISGHHHHHH*
**QAE(T18N)**-6xHIS	MGMNQASVVANQLIPINTALNLVMMRSEVVTPVGIPAEDIPRLVSMQVNRAVP<br>LGTTLMPDMVKGYAALEHHHHHH*
**HaloTag**-GAG-**QAE(T18N)**-6xHIS	MGAEIGTGFPFDPHYVEVLGERMHYVDVGPRDGTPVLFLHGNPTSSYVWRNII<br>PHVAPTHRCIAPDLIGMGKSDKPDLGYFFDDHVRFMDAFIEALGLEEVVLVIH<br>DWGSALGFHWAKRNPERVKGIAFMEFIRPIPTWDEWPEFARETFQAFRTTDVG<br>RKLIIDQNVFIEGTLPMGVVRPLTEVEMDHYREPFLNPVDREPLWRFPNELPI<br>AGEPANIVALVEEYMDWLHQSPVPKLLFWGTPGVLIPPAEAARLAKSLPNCKA<br>VDIGPGLNLLQEDNPDLIGSEIARWLSTLEISGEFGAGMNQASVVANQLIPIN<br>TALNLVMMRSEVVTPVGIPAEDIPRLVSMQVNRAVPLGTTLMPDMVKGYAALE<br>HHHHHH*
**mEos3.2**-GAG-**QAE(T18N)**-6xHIS	MGSAIKPDMKIKLRMEGNVNGHHFVIDGDGTGKPFEGKQSMDLEVKEGGPLPF<br>AFDILTTAFHYGNRVFAKYPDNIQDYFKQSFPKGYSWERSLTFEDGGICNARN<br>DITMEGDTFYNKVRFYGTNFPANGPVMQKKTLKWEPSTEKMYVRDGVLTGDIE<br>MALLLEGNAHYRCDFRTTYKAKEKGVKLPGAHFVDHCIEILSHDKDYNKVKLY<br>EHAVAHSGLPDNARREFGAGMNQASVVANQLIPINTALNLVMMRSEVVTPVGI<br>PAEDIPRLVSMQVNRAVPLGTTLMPDMVKGYAALEHHHHHH*
**QAE(A16R)**-6xHIS	MGMNQASVVANQLIPINTRLTLVMMRSEVVTPVGIPAEDIPRLVSMQVNRAVP<br>LGTTLMPDMVKGYAALEHHHHHH*
**HaloTag**-GAG-	MGMGAEIGTGFPFDPHYVEVLGERMHYVDVGPRDGTPVLFLHGNPTSSYVWRN

**QAE(A16R)**-6xHIS	IIPHVAPTHRCIAPDLIGMGKSDKPDLGYFFDDHVRFMDAFIEALGLEEVVLV<br>IHDWGSALGFHWAKRNPERVKGIAFMEFIRPIPTWDEWPEFARETFQAFRTTD<br>VGRKLIIDQNVFIEGTLPMGVVRPLTEVEMDHYREPFLNPVDREPLWRFPNEL<br>PIAGEPANIVALVEEYMDWLHQSPVPKLLFWGTPGVLIPPAEAARLAKSLPNC<br>KAVDIGPGLNLLQEDNPDLIGSEIARWLSTLEISGEFGAGMNQASVVANQLIP<br>INTRLTLVMMRSEVVTPVGIPAEDIPRLVSMQVNRAVPLGTTLMPDMVKGYAA<br>LEHHHHHH*
6xHIS-restriction_site-***Lp*IBP**	MGHHHHHHGSGASGSKLGSGDEQPNTISGSNNTVRSGSKNVLAGNDNTVISGD<br>NNSVSGSNNTVVSGNDNTVTGSNHVVSGTNHIVTDNNNNVSGNDNNVSGSFHT<br>VSGGHNTVSGSNNTVSGSNHVVSGSNKVVTD*
6xHIS-thrombin_site-**HaloTag**-GGGS-***Lp*IBP**-6xHIS	MGSSHHHHHHSSGLVPRGSHMMGAEIGTGFPFDPHYVEVLGERMHYVDVGPRD<br>GTPVLFLHGNPTSSYVWRNIIPHVAPTHRCIAPDLIGMGKSDKPDLGYFFDDH<br>VRFMDAFIEALGLEEVVLVIHDWGSALGFHWAKRNPERVKGIAFMEFIRPIPT<br>WDEWPEFARETFQAFRTTDVGRKLIIDQNVFIEGTLPMGVVRPLTEVEMDHYR<br>EPFLNPVDREPLWRFPNELPIAGEPANIVALVEEYMDWLHQSPVPKLLFWGTP<br>GVLIPPAEAARLAKSLPNCKAVDIGPGLNLLQEDNPDLIGSEIARWLSTLEIS<br>GGGGSGGGSMDEQPNTISGSNNTVRSGSKNVLAGNDNTVISGDNNSVSGSNNT<br>VVSGNDNTVTGSNHVVSGTNHIVTDNNNNVSGNDNNVSGSFHTVSGGHNTVSG<br>SNNTVSGSNHVVSGSNKVVTDAAKLAAALEHHHHHH*
**wfAFP**	DTASDAAAAAALTAANAKAAAELTAANAAAAAAATAR
**HaloTag-**GGGGS-**wfAFP**-6xHIS	MGAEIGTGFPFDPHYVEVLGERMHYVDVGPRDGTPVLFLHGNPTSSYVWRNII<br>PHVAPTHRCIAPDLIGMGKSDKPDLGYFFDDHVRFMDAFIEALGLEEVVLVIH<br>DWGSALGFHWAKRNPERVKGIAFMEFIRPIPTWDEWPEFARETFQAFRTTDVG<br>RKLIIDQNVFIEGTLPMGVVRPLTEVEMDHYREPFLNPVDREPLWRFPNELPI<br>AGEPANIVALVEEYMDWLHQSPVPKLLFWGTPGVLIPPAEAARLAKSLPNCKA<br>VDIGPGLNLLQEDNPDLIGSEIARWLSTLEISGEFGGGGSDTASDAAAAAALT<br>AANAKAAAELTAANAAAAAAATARLEHHHHHH*
6xHIS-thrombin_site-**HaloTag**-GAG-6xHIS	MGSSHHHHHHSSGLVPRGSHMMGAEIGTGFPFDPHYVEVLGERMHYVDVGPRD<br>GTPVLFLHGNPTSSYVWRNIIPHVAPTHRCIAPDLIGMGKSDKPDLGYFFDDH<br>VRFMDAFIEALGLEEVVLVIHDWGSALGFHWAKRNPERVKGIAFMEFIRPIPT<br>WDEWPEFARETFQAFRTTDVGRKLIIDQNVFIEGTLPMGVVRPLTEVEMDHYR<br>EPFLNPVDREPLWRFPNELPIAGEPANIVALVEEYMDWLHQSPVPKLLFWGTP<br>GVLIPPAEAARLAKSLPNCKAVDIGPGLNLLQEDNPDLIGSEIARWLSTLEIS<br>GEFGAGHHHHHH*
6xHIS-thrombin_site-**HaloTag**-GAG-**SUMO-**6xHIS	MGSSHHHHHHSSGLVPRGSHMMGAEIGTGFPFDPHYVEVLGERMHYVDVGPRD<br>GTPVLFLHGNPTSSYVWRNIIPHVAPTHRCIAPDLIGMGKSDKPDLGYFFDDH<br>VRFMDAFIEALGLEEVVLVIHDWGSALGFHWAKRNPERVKGIAFMEFIRPIPT<br>WDEWPEFARETFQAFRTTDVGRKLIIDQNVFIEGTLPMGVVRPLTEVEMDHYR<br>EPFLNPVDREPLWRFPNELPIAGEPANIVALVEEYMDWLHQSPVPKLLFWGTP<br>GVLIPPAEAARLAKSLPNCKAVDIGPGLNLLQEDNPDLIGSEIARWLSTLEIS<br>GEFGAGDSEVNQEAKPEVKPEVKPETHINLKVSDGSSEIFFKIKKTTPLRRLM<br>EAFAKRQGKEMDSLRFLYDGIRIQADQTPEDLDMEDNDIIEAHREQIGGLEHH<br>HHHH*
**EGF(epidermal growth factor)**-GGGSGGGS-**HaloTag**	MGEFHMNSDSECPLSHDGYCLHDGVCMYIEALDKYACNCVVGYIGERCQYRDL<br>KWWELRGGGSGGGSMAEIGTGFPFDPHYVEVLGERMHYVDVGPRDGTPVLFLH<br>GNPTSSYVWRNIIPHVAPTHRCIAPDLIGMGKSDKPDLGYFFDDHVRFMDAFI<br>EALGLEEVVLVIHDWGSALGFHWAKRNPERVKGIAFMEFIRPIPTWDEWPEFA<br>RETFQAFRTTDVGRKLIIDQNVFIEGTLPMGVVRPLTEVEMDHYREPFLNPVD<br>REPLWRFPNELPIAGEPANIVALVEEYMDWLHQSPVPKLLFWGTPGVLIPPAE<br>AARLAKSLPNCKAVDIGPGLNLLQEDNPDLIGSEIARWLSTLEISGHHHHHH*

Table S2. Protein data bank (PDB) codes of proteins in this work

**HaloTag**	6U32
**mEos3.2**	9J11
**SNAPtag**	6Y8P
**QAE**	1HZG
**wfAFP**	1WFA
***Lp*IBP**	3ULT
**SUMOtag**	1L2N
**EGF**	2KV4

Protein expression and purification of HaloTag constructs

In view of the surprising findings regarding the effect of HaloTag on IRI activity, we performed additional assessments with the fusion constructs with HaloTag and the separate HaloTag. These protein constructs were expressed and purified multiple times to evaluate the reproducibility of ice-binding activity, and the purity was determined with SDS-PAGE gels (Figure S6) For HaloTag additional purity analysis was determined with QToF mass spectrometry (Figure S7). For the fusion constructs HaloTag-QAE, HaloTag-QAE(A16R) and HaloTag-QAE(T18N), protein from multiple expression rounds showed one order of magnitude enhancement in IRI activity compared to the protein without tag. However, the separate HaloTag only showed IRI activity in the first round of expression. As the QToF spectrum of this sample (Figure S7) indicates highly pure protein with the expected size, we concluded that HaloTag is susceptible to small structural changes during the expression or purification process, which can diminish its ability to interact with ice.

Figure S6 SDS-PAGE gels of the expressed protein constructs. During the purification process several samples were taken. In case of significant impurities after the first IMAC purification, the sample was further purified with a second IMAC column. **cult** = culture sample immediately after expression. **sup** = supernatant after lysis and centrifugation **ft** = flow through from IMAC column after applying the supernatant **wash** = wash fraction from IMAC column after applying wash buffer **elut** = protein sample eluted from the desalting column (x times diluted on the gel depending on the protein concentration).

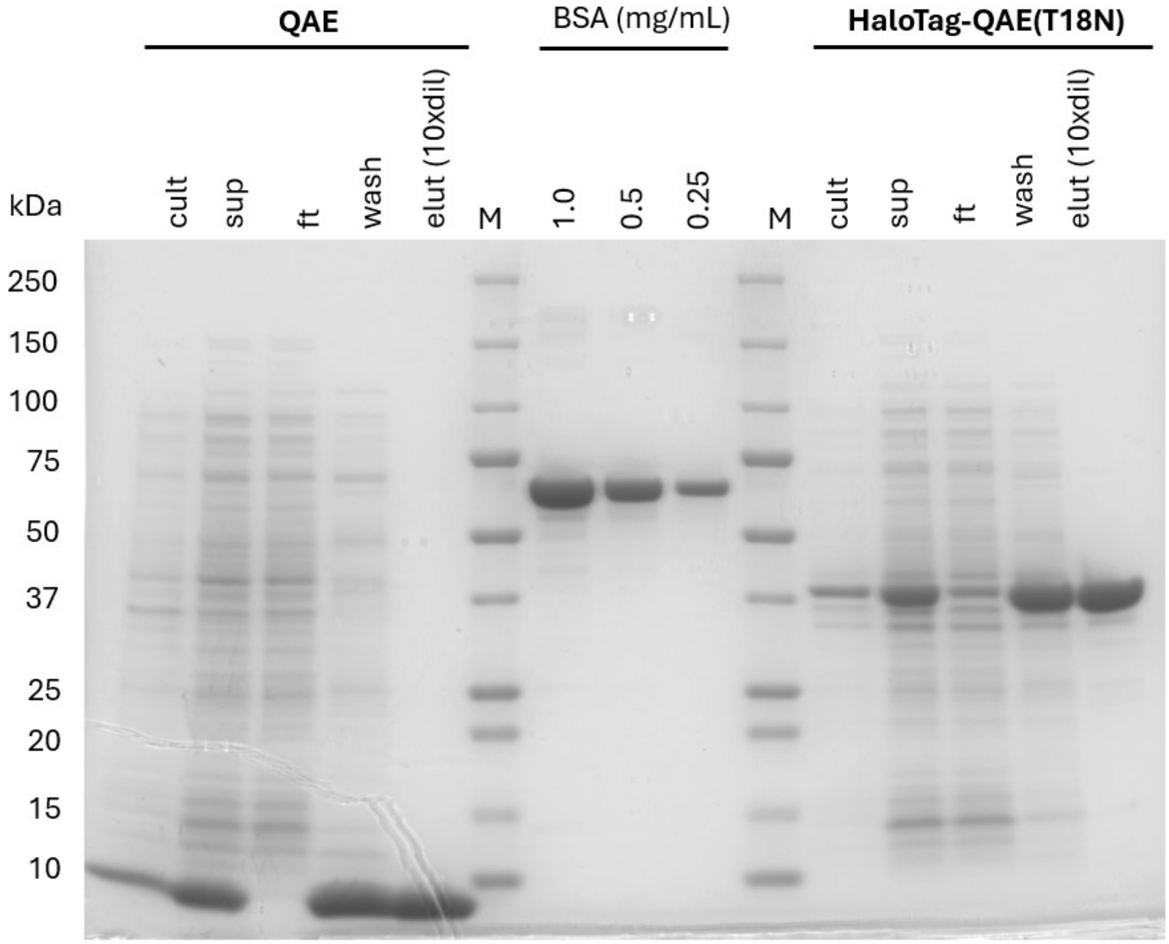

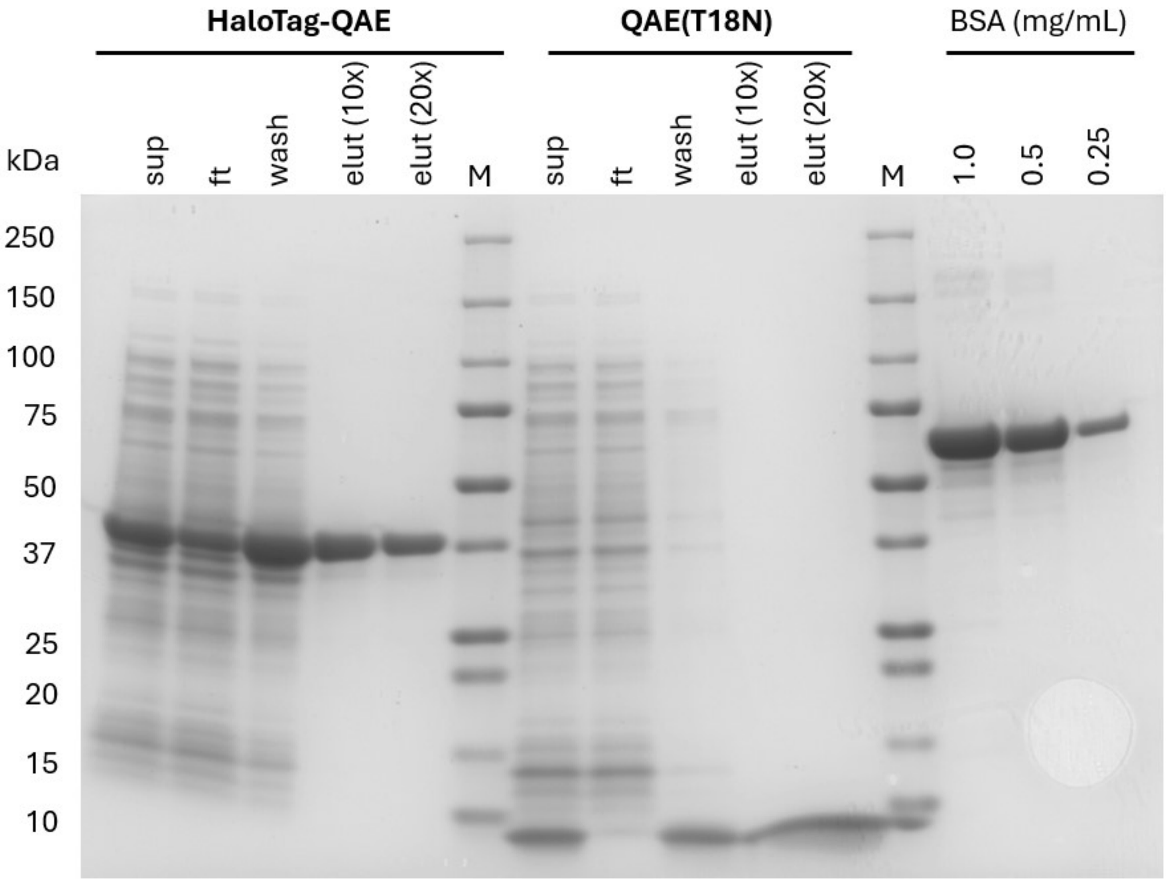
HaloTag-QAE
QAE(T18N)
BSA (mg/mL)
kDa
sup
ft
wash
elut (10x)
elut (20x)
M
sup
ft
wash
elut (10x)
elut (20x)
M
1.0
0.5
0.25
250
150
100
75
50
37
25
20
15
10

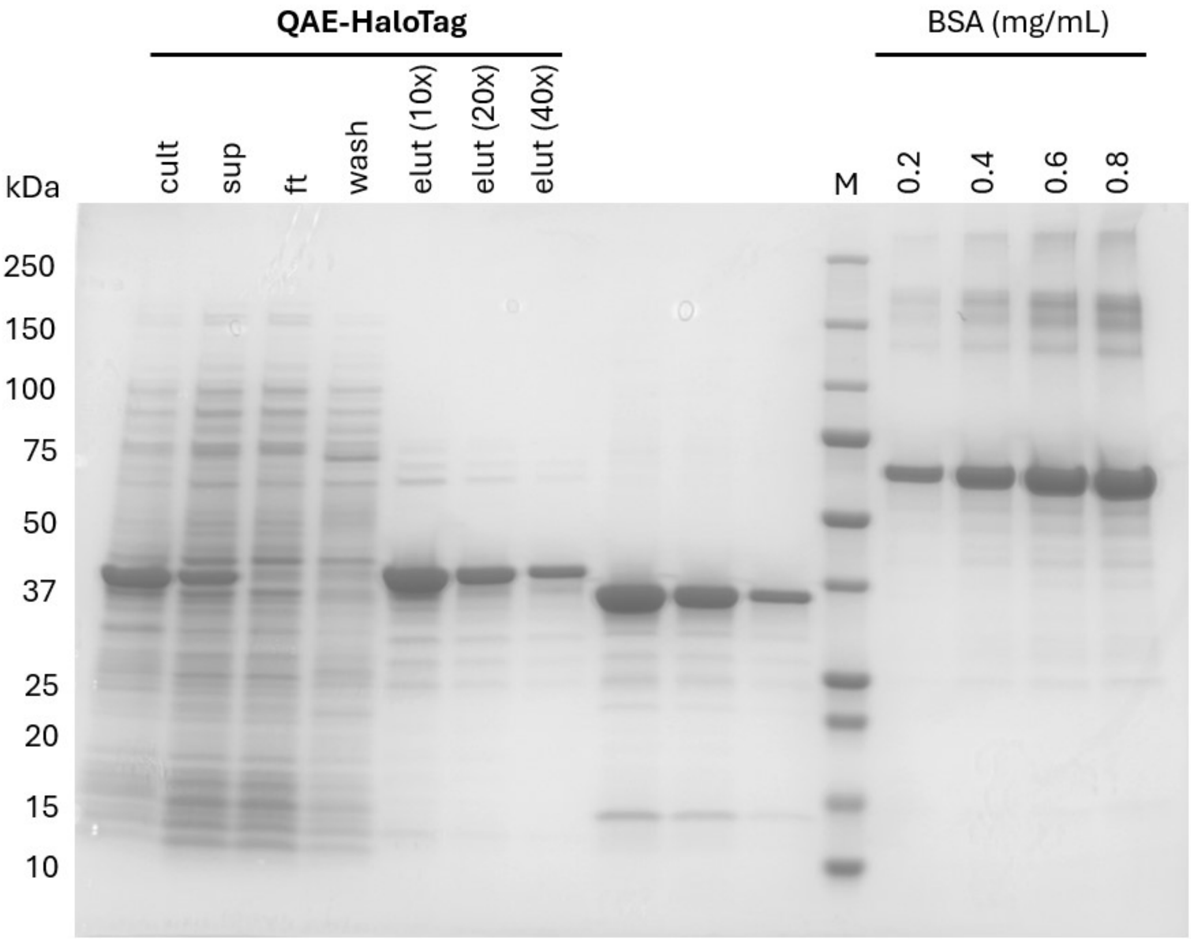
QAE-HaloTag
BSA (mg/mL)
kDa
cult
sup
ft
wash
elut (10x)
elut (20x)
elut (40x)
M
0.2
0.4
0.6
0.8
250
150
100
75
50
37
25
20
15
10

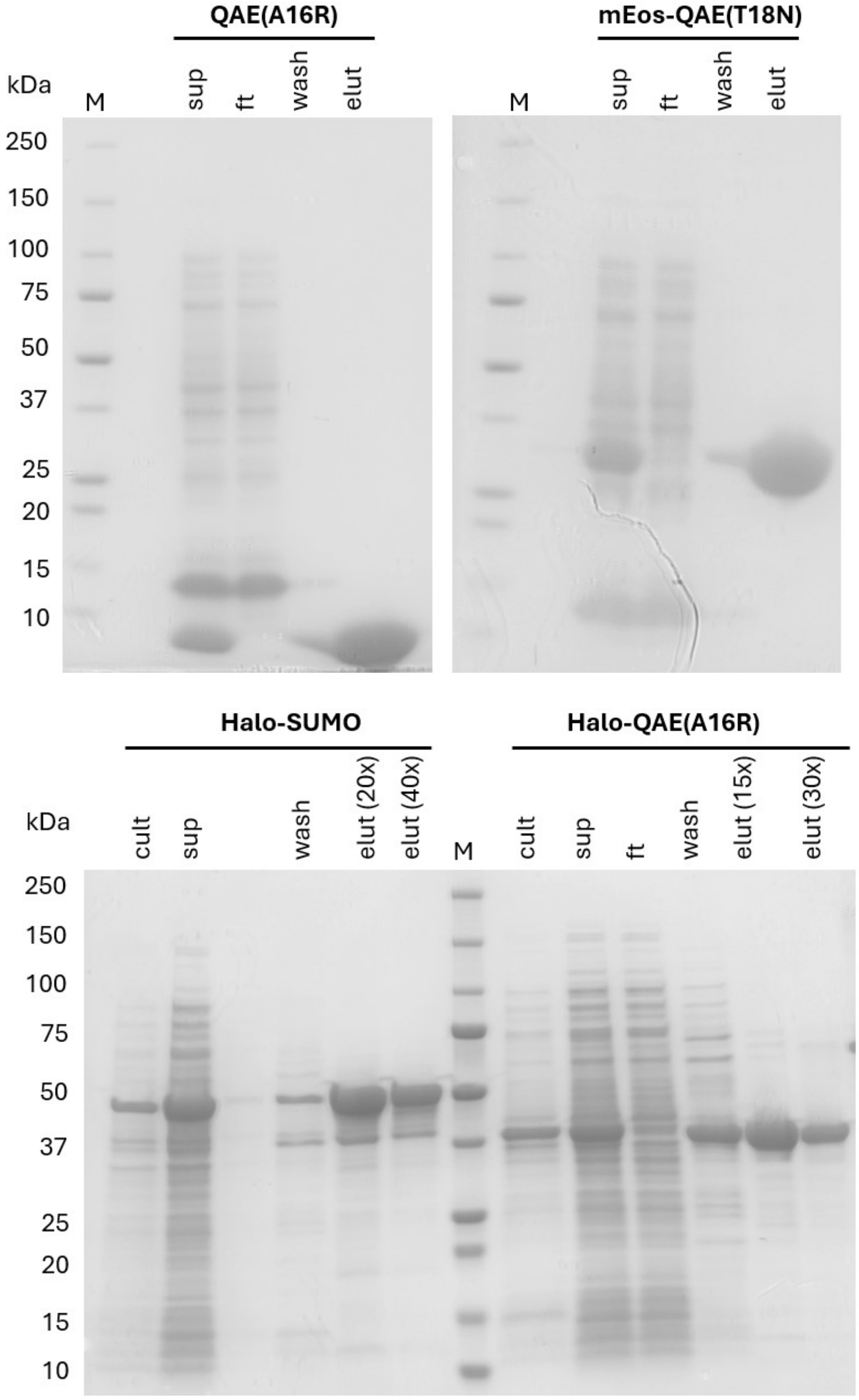
QAE(A16R)
mEos-QAE(T18N)
kDa
M
sup
ft
wash
elut
M
sup
ft
wash
elut
250
150
100
75
50
37
25
20
15
10
Halo-SUMO
Halo-QAE(A16R)
kDa
cult
sup
wash
elut (20x)
elut (40x)
M
cult
sup
ft
wash
elut (15x)
elut (30x)
250
150
100
75
50
37
25
20
15
10

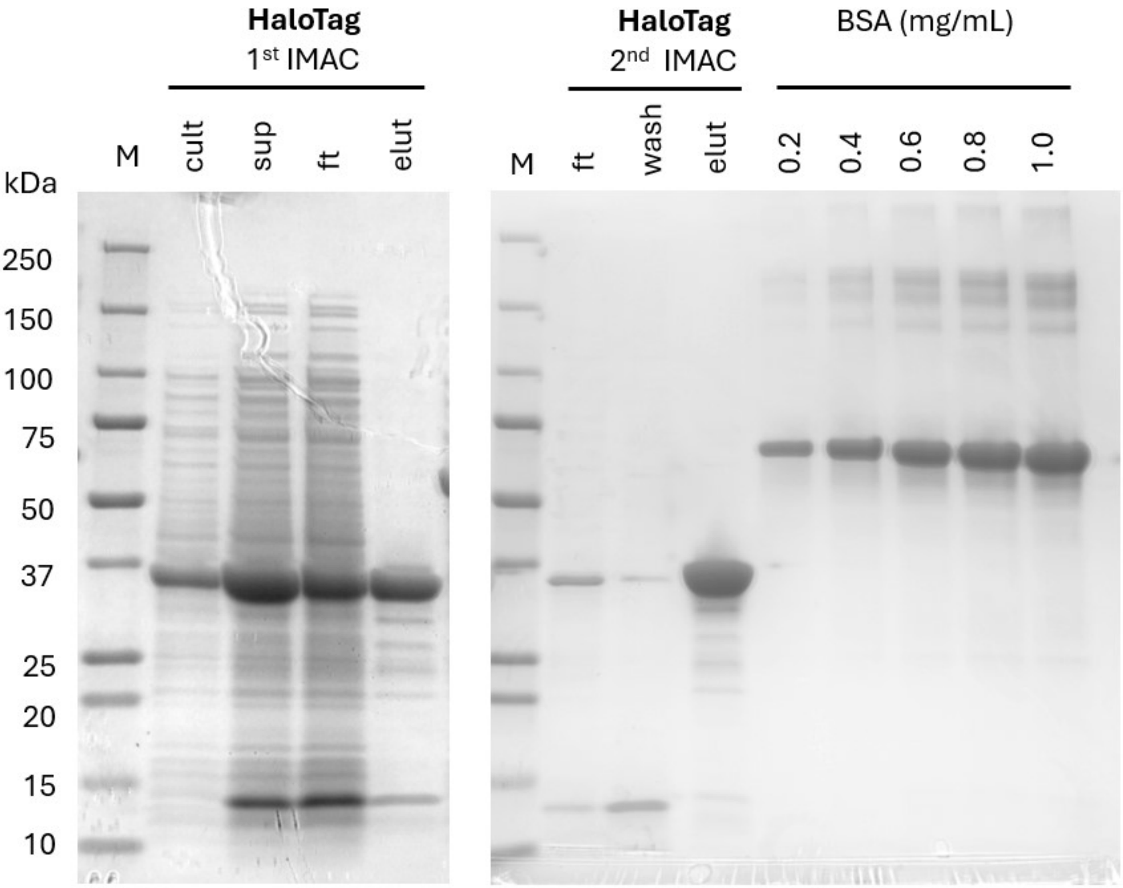
HaloTag
1st IMAC
HaloTag
2nd IMAC
BSA (mg/mL)
M
cult
sup
ft
elut
M
ft
wash
elut
0.2
0.4
0.6
0.8
1.0
kDa
250
150
100
75
50
37
25
20
15
10

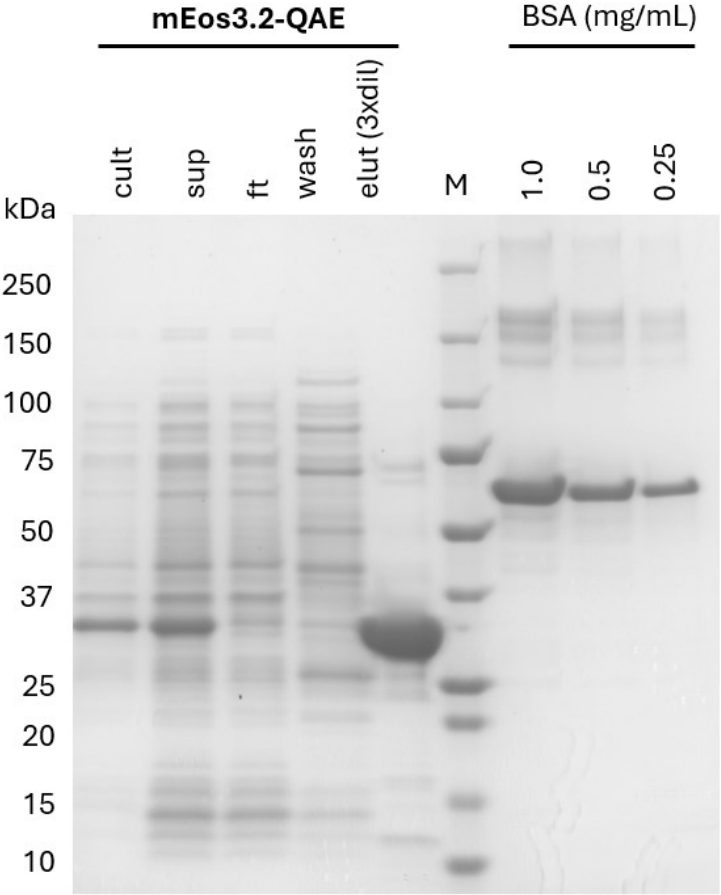
mEos3.2-QAE
BSA (mg/mL)
cult
sup
ft
wash
elut (3xdil)
M
1.0
0.5
0.25
kDa
250
150
100
75
50
37
25
20
15
10

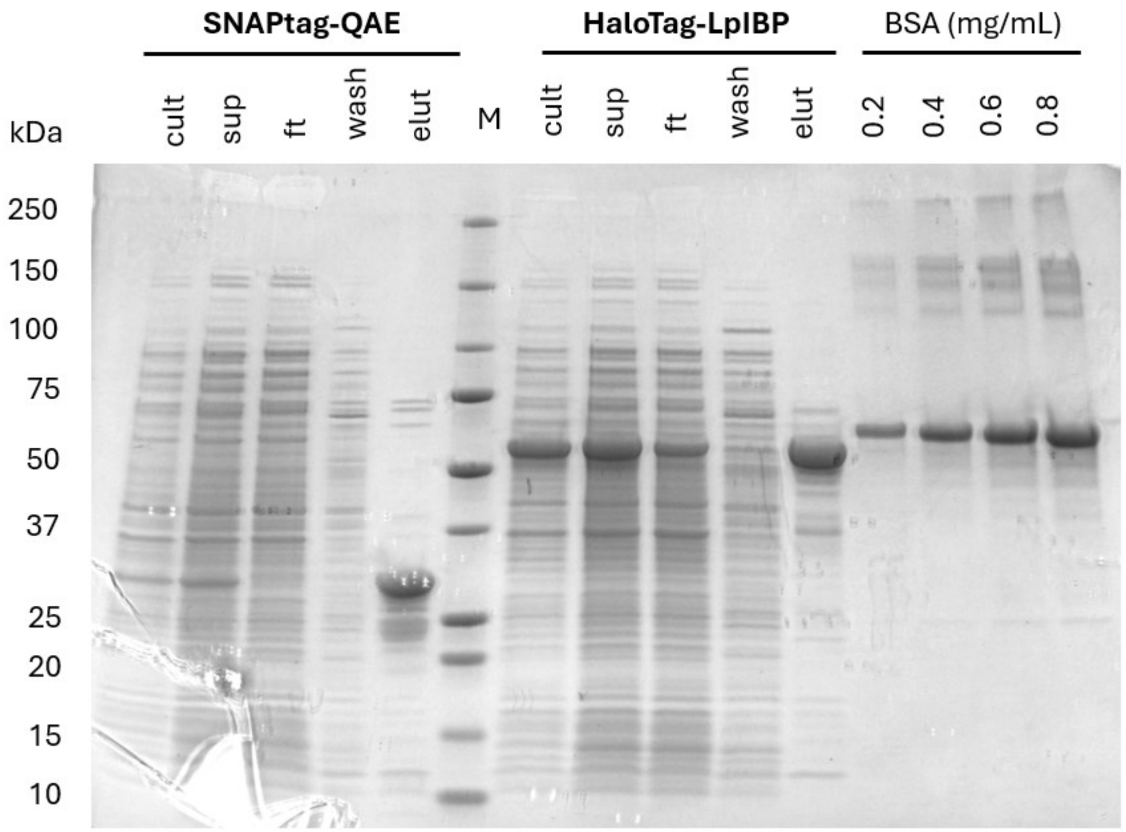
SNAPtag-QAE
HaloTag-LpIBP
BSA (mg/mL)
kDa
cult
sup
ft
wash
elut
M
cult
sup
ft
wash
elut
0.2
0.4
0.6
0.8
250
150
100
75
50
37
25
20
15
10

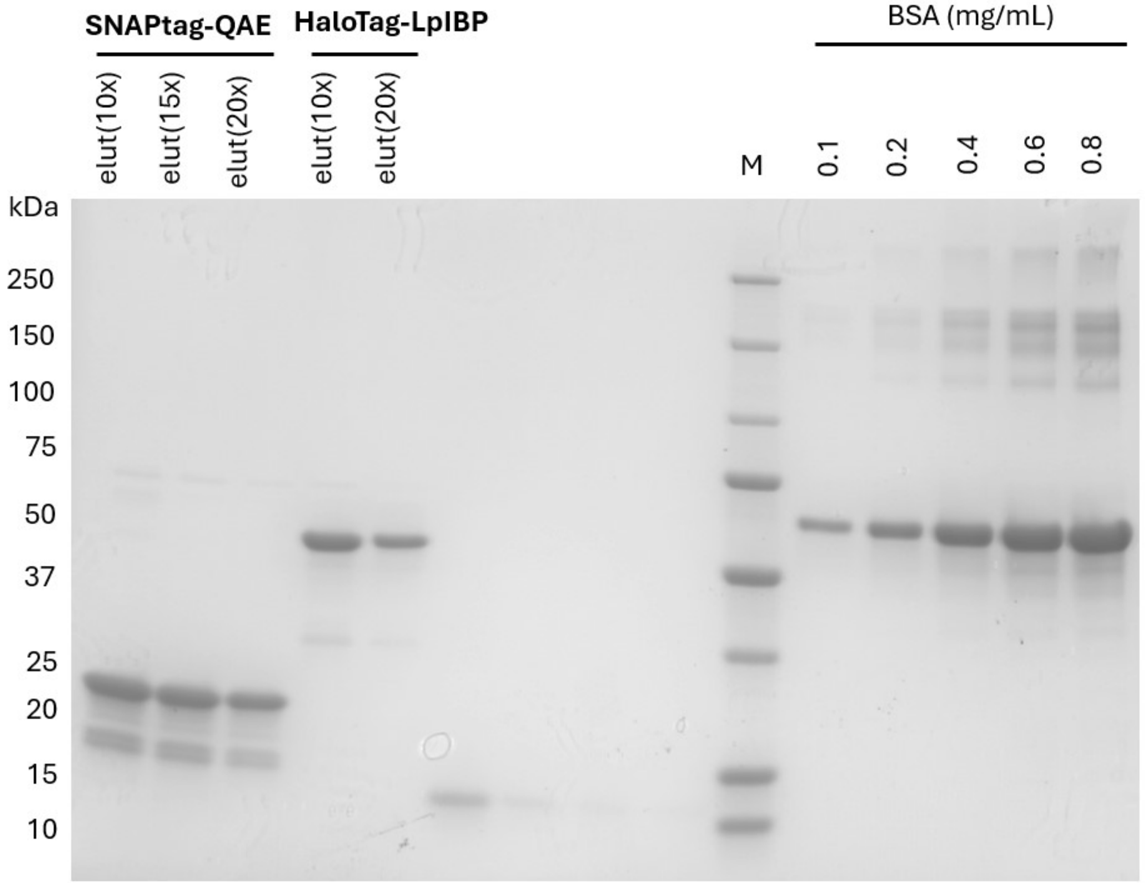
SNAPtag-QAE
HaloTag-LpIBP
BSA (mg/mL)
elut(10x)
elut(15x)
elut(20x)
elut(10x)
elut(20x)
M
0.1
0.2
0.4
0.6
0.8
kDa
250
150
100
75
50
37
25
20
15
10

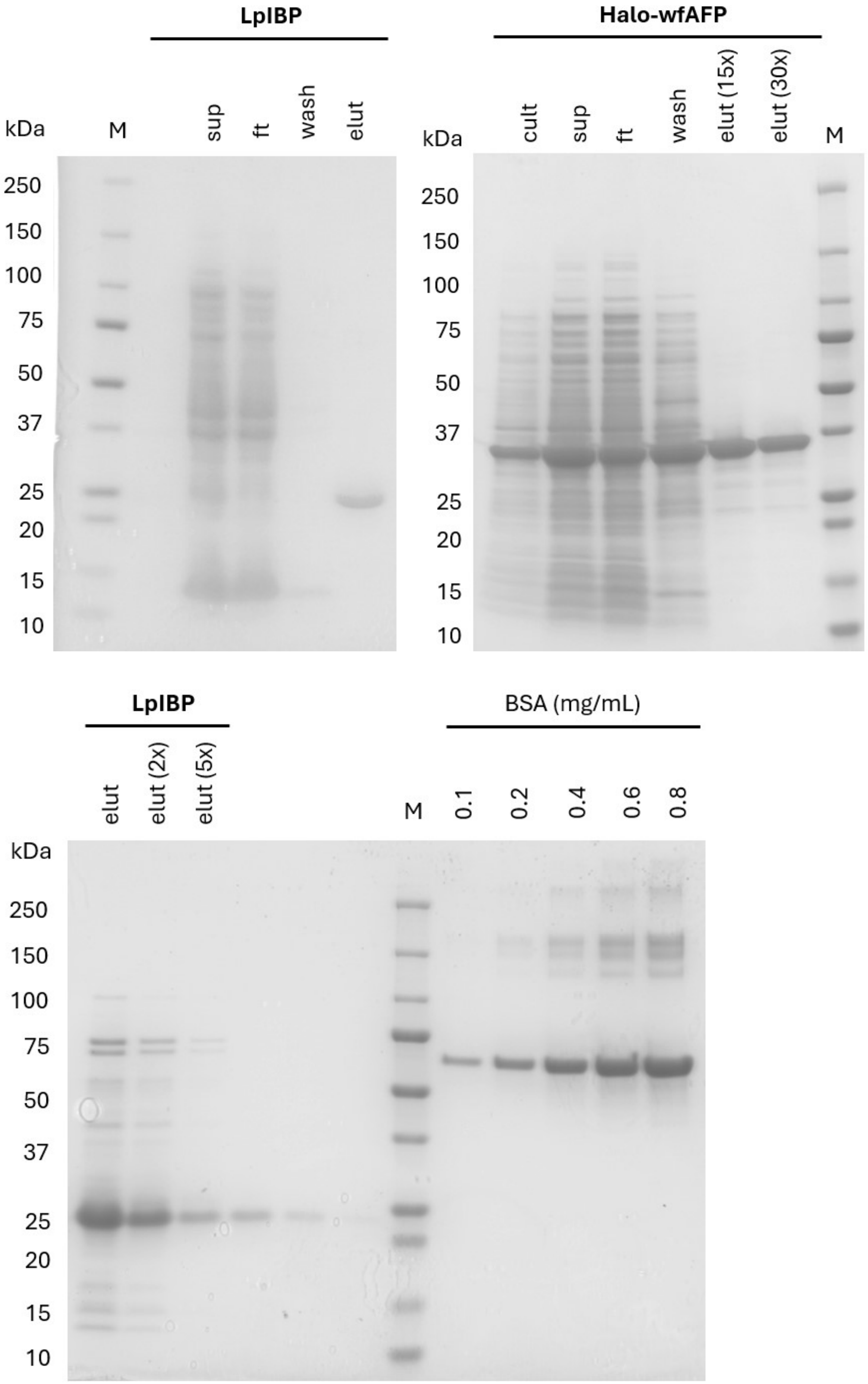
LpIBP
Halo-wfAFP
kDa
M
sup
ft
wash
elut
cult
sup
ft
wash
elut (15x)
elut (30x)
M
250
150
100
75
50
37
25
20
15
10
LpIBP
BSA (mg/mL)
elut
elut (2x)
elut (5x)
M
0.1
0.2
0.4
0.6
0.8

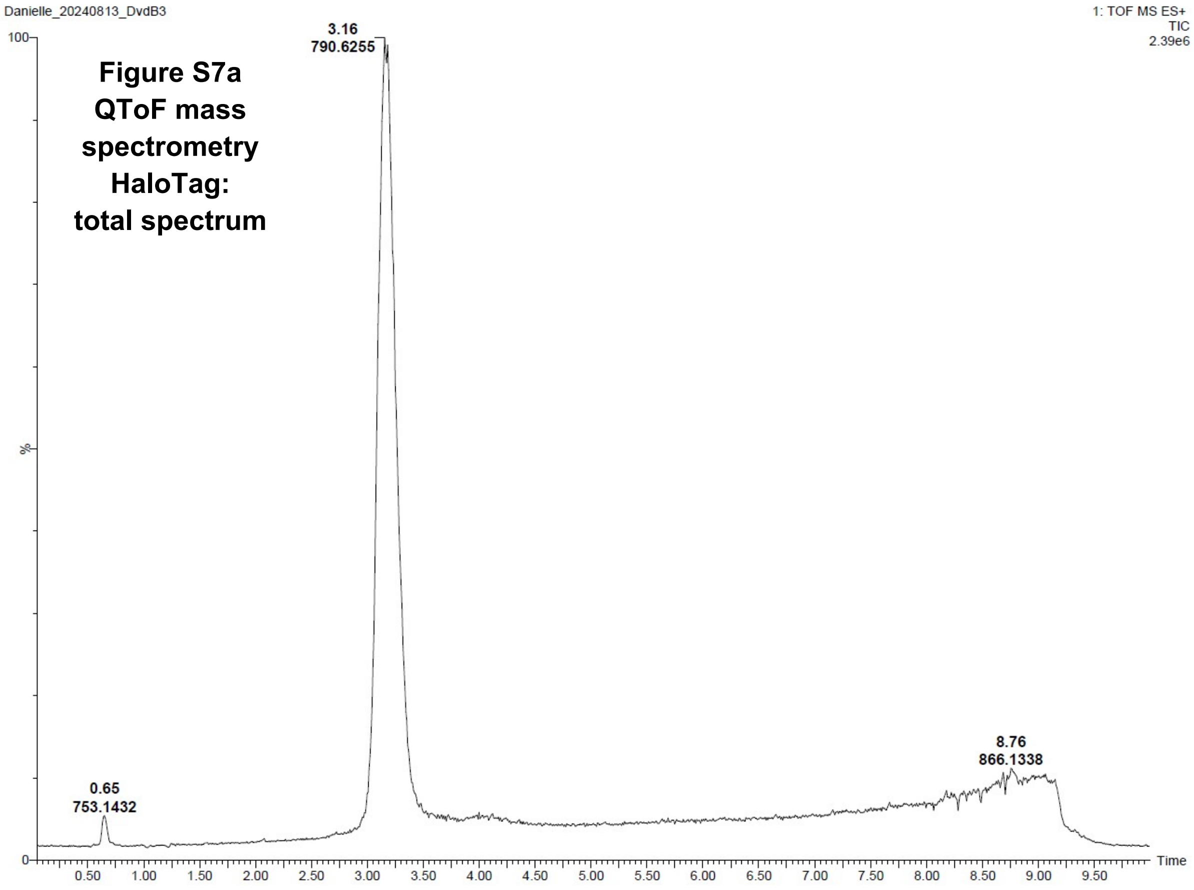
Danielle_20240813_DvdB3
1: TOF MS ES+
TIC
2.39e6
Figure S7a
QToF mass
spectrometry
HaloTag:
total spectrum
3.16
790.6255
0.65
753.1432
8.76
866.1338
100
%
0
0.50 1.00 1.50 2.00 2.50 3.00 3.50 4.00 4.50 5.00 5.50 6.00 6.50 7.00 7.50 8.00 8.50 9.00 9.50
Time

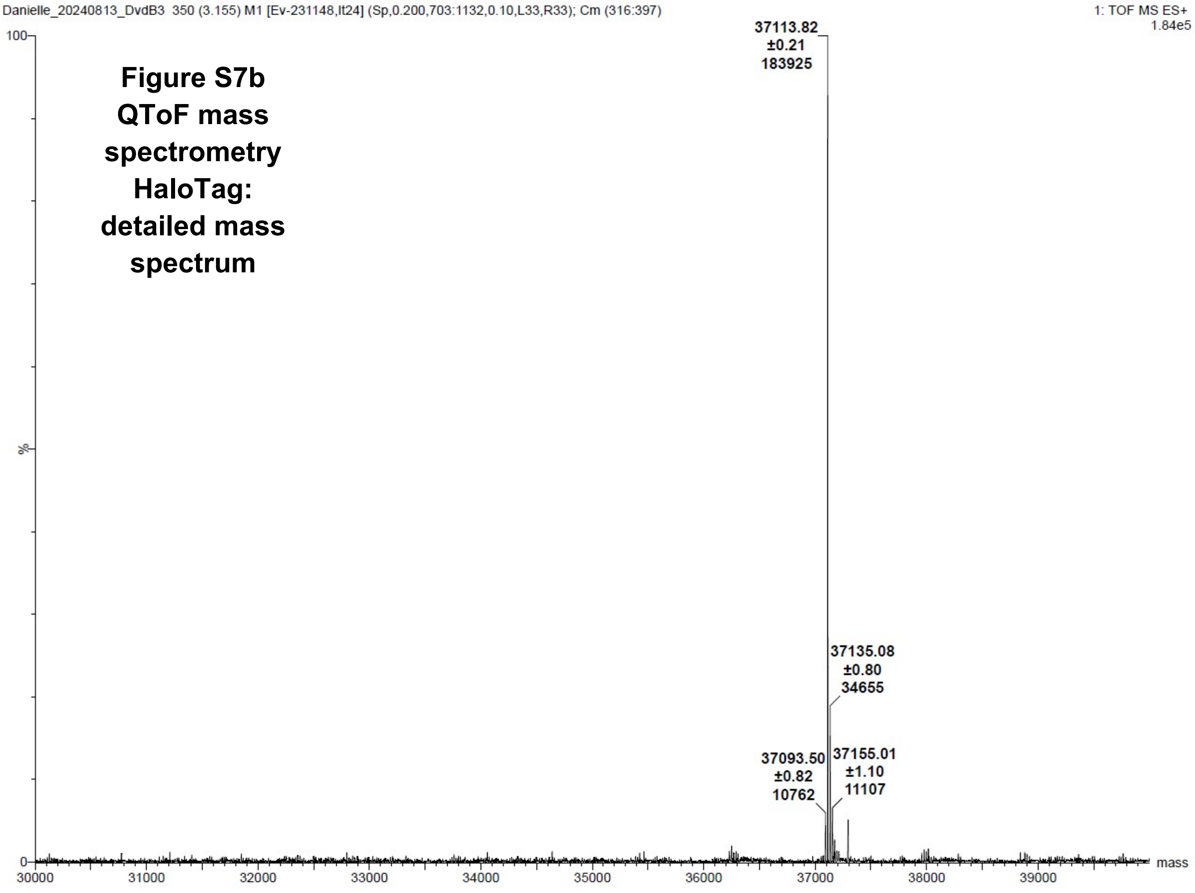
Danielle_20240813_DvdB3 350 (3.155) M1 [Ev-231148,It24] (Sp,0.200,703:1132,0.10,L33,R33); Cm (316:397)
1: TOF MS ES+
1.84e5
Figure S7b
QToF mass
spectrometry
HaloTag:
detailed mass
spectrum
37113.82
±0.21
183925
37135.08
±0.80
34655
37093.50
±0.82
10762
37155.01
±1.10
11107
100
%
0
30000 31000 32000 33000 34000 35000 36000 37000 38000 39000
mass

4. Solid phase peptide synthesis

Methods: Synthesis of AFGP analogue (23,24)

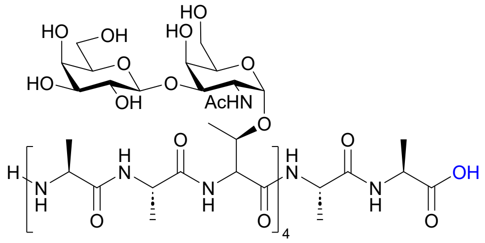

$C_{102}H_{172}N_{18}O_{59}$

MW:2594.57

$[C_{102}H_{172}N_{18}O_{59}+H]^+$: 2595.11155

$[C_{102}H_{172}N_{18}O_{59}+Na]^+$: 2617.09349

$[C_{102}H_{172}N_{18}O_{59}+K]^+$: 2633.06743

The glycosylated threonine amino acid (*O*-[2-Acetamido-4,6-di-*O*-acetyl-2-deoxy-3-*O*-(2,3,4,6-tetra-*O*-acetyl-β-D-galactopyranosyl)-α-D-galactopyranosyl]-*N*-[(9*H*-fluoren-9-yl)-methoxycarbonyl]-L-threonine) was synthesized inhouse. All other Fmoc-amino acids, HBTU, HOAt, DIPEA and resins were purchased from Sigma-Aldrich (Taufkirchen, Germany) or Iris Biotech GmbH (Marktredwitz, Germany). DMF, MeCN and piperidine were purchased from Biosolve BV (Valkenswaard, The Netherlands) and used without additional purification.

The glycopeptide was synthesized on Fmoc-L-Ala AC TentaGel (0.23 mmol/g, 0.1 mmol, Iris Biotech SAL1101.0005), following the Fmoc/t-Bu strategy. The sequence elongation was performed on a 0.1 mmol scale on a microwave-assisted solid-phase peptide synthesizer (Liberty Blue, CEM, Matthews, NC, U.S.A.). The reaction temperatures were monitored by an internal fiber-optic sensor and deprotection and coupling reactions were performed in a Teflon vessel, applying microwave energy under nitrogen bubbling.

The peptide elongation was performed by repeating the MW-SPPS cycle for each amino acid. In short peptides were elongated with the following cycle. Fmoc deprotection was performed using a 20% (v/v) piperidine solution in DMF in 2 steps: 40°C, 30W, 30s, ΔT=0°C; 40°C, 10W, 270s, ΔT=0°C, followed by washing with DMF (3x 4mL). Coupling was performed in 3 steps 25°C, 2W, 30s, ΔT=2°C; 50°C, 50W, 30s, ΔT=0°C; 50°C, 15W, 570s, ΔT=0°C, see below for quantities per amino acid. The MW-SPPS cycle was finished with a DMF wash step (4mL). Coupling of per-acetylated GalGlcNAC functionalized Fmoc-L-threonine (2 eq, 0.1 M in DMF) was achieved by activation with HBTU (1.8 eq, 0.18 M in DMF), HOAt (1.8 eq, 0.18 M in DMF) and DIPEA (5 eq, 0.5 M in DMF). All other couplings were performed with 5 eq. of amino acid that were activated with HBTU (4.5 eq, 0.45 M in DMF), HOAt (4 eq, 0.40 M in DMF) and DIPEA (10 eq, 1.0 M in DMF). Once the synthesis was finished, the resin was transferred into a syringe equipped with a filter, washed with DMF (3x4mL), DCM (3x4mL) and dried under nitrogen flow.

The resin was resuspended and swollen in DMF (2x4mL), treated 40 minutes with hydrazine monohydrate in DMF (4mL of a 1.6 mL hydrazine monohydrate solution in 20 mL DMF) while shaking. This was repeated four times in total. The resin was washed with DMF (4 x 4mL) and DCM (5x 4mL), and the glycopeptide was released by treatment with DCM/HFIP/TIS (5 mL, 28/12/1, v/v/v), while shaking. The filtrate was dripped in cold Et2O/hexane (45mL, 1/1, v/v) and spinned down. The pellet was redissolved in MQ and lyophilized to yield the crude glycopeptide as a white powder. Purification via RP-HPLC (rt = 20min, linear gradient of 0% -> 30% of B in A over 60 minutes, 4 mL/min, ReproSil Gold 120 C18 5µm - 250 x 10 mm, with eluent A = TFA in MQ (1/999, v/v) and eluent B = TFA in MeCN (1/999, v/v)), yielded glycopeptide as a white powder after lyophilization (9.79 mg, 3.77µmol, 3.8%) confirmed by NMR and MALDI-TOF.

Methods: Synthesis of wfAFP

wfAFP sequence: *H*-DTASDAAAAAALTAANAKA AAELTAANAAAAAAATAR-*NH*$_2$

$C_{133}H_{226}N_{44}O_{50}$

MW: 3242.53

$[C_{133}H_{226}N_{44}O + H]^{+}$: 3241.6595;

$[C_{133}H_{226}N_{44}O_{50} + 2H]^{2+}$: 1621.3334

$[C_{133}H_{226}N_{44}O_{50} + 3H]^{3+}$: 1081.2247

$[C_{133}H_{226}N_{44}O_{50} + 4H]^{4+}$: 811.1703

The *wf*AFP was synthesized on TentaGel™ S-RAM resin (loading 0.25 mmol/g, 100 µm), following the Fmoc/t-Bu strategy under nitrogen atmosphere. The sequence elongation was performed on a 0.1 mmol scale with a fivefold molar excess of Fmoc-AA-OH on a microwave-assisted solid-phase peptide synthesizer (Liberty Blue, CEM, Matthews, NC, U.S.A.). Activation was achieved with DIC and Oxyma Pure at 90 °C. The reaction temperatures were monitored by an internal fiber-optic sensor and deprotection and coupling reactions were performed in a Teflon vessel, applying microwave energy under nitrogen bubbling. The peptide elongation was performed by repeating the MW-SPPS cycle for each amino acid, using a modified protocol based on the CARBOMAX method provided by CEM.

In short, the peptide was elongated with the following cycles. Fmoc deprotection was performed using a 20% (v/v) piperidine solution in DMF in four steps: 25 °C, 2 W, 30 s, ΔT = 5 °C; 75 °C, 125 W, 20 s, ΔT = 1 °C; 90 °C, 5 W, 5 s, ΔT = 0 °C; 90 °C, 20 W, 90 s, ΔT = 0 °C, followed by washing with DMF (3 × 4 mL). Coupling was performed with Fmoc-AA-OH (2.5 mL, 0.2 M in DMF, 0.5 mmol, 5 eq), DIC (1 mL, 1.0 M in DMF, 1 mmol, 10 eq), and Oxyma Pure (0.5 mL, 1.0 M in DMF, 0.5 mmol, 5 eq) in 4 steps: 40 °C, 5 W, 30 s, ΔT = 5 °C; 75 °C, 163 W, 12 s, ΔT = 1 °C; 90 °C, 5 W, 9 s, ΔT = 0 °C; 90 °C, 20 W, 120 s, ΔT = 0 °C, followed by a DMF wash step (4 mL). All couplings after β-branched amino acids (in bold) used prolonged coupling times of 240 s for the final step.

Once the synthesis was complete, the resin was transferred into a syringe equipped with a filter, washed with DMF (3 × 4 mL), DCM (3 × 4 mL), and dried under nitrogen flow. Crude peptide was obtained after acidic deprotection and cleavage from resin with TFA/TIS/H_2O

(10 mL; 190/5/5; v/v/v) for 2 h while shaking, followed by precipitation in cold Et_2O (50 mL). Purification by reverse-phase high-performance liquid chromatography (linear-gradient 10 to 70% ACN in water with 0.1% TFA, 60 min, ReproSil Gold 120 C18, 10 μm–240 × 20 mm, 5 mL/min) yielded wfAFP as a white powder after lyophilization in 8.59 mg (2.65 μmol). HPLC-MS analysis was performed to validate purity, using a C18 Jupiter SuC4300A 150 × 2.0 mm column using miliQ water with 0.1% FA and acetonitrile with 0.1% FA, using a gradient of 5 to 100% ACN over 10 min, connected to a Thermo Fisher LTQ XL Linear Ion Trap Mass Spectrometer (rt = 3.25 min).

5. References

1. Braslavsky I, Drori R. LabVIEW-operated Novel Nanoliter Osmometer for Ice Binding Protein Investigations. J Vis Exp. 2013 Feb 4;(72):4189.

2. Pariente N, Bar Dolev M, Braslavsky I. The Nanoliter Osmometer: Thermal Hysteresis Measurement. In: Drori R, Stevens C, editors. Ice Binding Proteins [Internet]. New York, NY: Springer US; 2024 [cited 2025 Mar 31]. p. 75–91. (Methods in Molecular Biology; vol. 2730). Available from: https://link.springer.com/10.1007/978-1-0716-3503-2_5

3. Knight CA, Cheng CC, DeVries AL. Adsorption of alpha-helical antifreeze peptides on specific ice crystal surface planes. Biophys J. 1991 Feb;59(2):409–18.

4. Basu K, Garnham CP, Nishimiya Y, Tsuda S, Braslavsky I, Davies P. Determining the ice-binding planes of antifreeze proteins by fluorescence-based ice plane affinity. J Vis Exp. 2014 Jan 15;(83).

5. Pertaya N, Marshall CB, DiPrinzio CL, Wilen L, Thomson ES, Wettlaufer JS, et al. Fluorescence Microscopy Evidence for Quasi-Permanent Attachment of Antifreeze Proteins to Ice Surfaces. Biophys J. 2007 May;92(10):3663–73.

6. Pertaya N, Marshall CB, Celik Y, Davies PL, Braslavsky I. Direct Visualization of Spruce Budworm Antifreeze Protein Interacting with Ice Crystals: Basal Plane Affinity Confers Hyperactivity. Biophys J. 2008 July;95(1):333–41.

7. Celik Y, Drori R, Pertaya-Braun N, Altan A, Barton T, Bar-Dolev M, et al. Microfluidic experiments reveal that antifreeze proteins bound to ice crystals suffice to prevent their growth. Proc Natl Acad Sci. 2013 Jan 22;110(4):1309–14.

8. Drori R, Celik Y, Davies PL, Braslavsky I. Ice-binding proteins that accumulate on different ice crystal planes produce distinct thermal hysteresis dynamics. J R Soc Interface. 2014 Sept 6;11(98):20140526.

9. Drori R, Davies PL, Braslavsky I. When Are Antifreeze Proteins in Solution Essential for Ice Growth Inhibition? Langmuir. 2015 June 2;31(21):5805–11.

10. Drori R, Davies PL, Braslavsky I. Experimental correlation between thermal hysteresis activity and the distance between antifreeze proteins on an ice surface. RSC Adv. 2015;5(11):7848–53.

11. Haleva L, Celik Y, Bar-Dolev M, Pertaya-Braun N, Kaner A, Davies PL, et al. Microfluidic Cold-Finger Device for the Investigation of Ice-Binding Proteins. Biophys J. 2016 Sept;111(6):1143–50.

12. Meister K, DeVries AL, Bakker HJ, Drori R. Antifreeze Glycoproteins Bind Irreversibly to Ice. J Am Chem Soc. 2018 Aug 1;140(30):9365–8.

13. Kaleda A, Haleva L, Sarusi G, Pinsky T, Mangiagalli M, Bar Dolev M, et al. Saturn-Shaped Ice Burst Pattern and Fast Basal Binding of an Ice-Binding Protein from an Antarctic Bacterial Consortium. Langmuir. 2019 June 11;35(23):7337–46.

14. Berger T, Meister K, DeVries AL, Eves R, Davies PL, Drori R. Synergy between Antifreeze Proteins Is Driven by Complementary Ice-Binding. J Am Chem Soc. 2019 Dec 4;141(48):19144–50.

15. Thosar AU, Shalom Y, Braslavsky I, Drori R, Patel AJ. Accumulation of Antifreeze Proteins on Ice Is Determined by Adsorption. J Am Chem Soc. 2023 Aug 16;145(32):17597–602.

16. Hobbs PV. Ice physics [Internet]. Saunders College Publishing; 1974 [cited 2025 Jan 27]. Available from: https://www.cambridge.org/core/product/identifier/S0022143000030847/type/journal_article

17. Owen CC, Hendrikse H. An initial study of interference coloration for quantifying the texture and fabric of ice. Cold Reg Sci Technol. 2023 Feb;206:103735.

18. Bloss FD. An Introduction to the Methods of Optical Crystallography [Internet]. Oxford: Clarendon Press; 1961 [cited 2025 Jan 27]. Available from: https://www.journals.uchicago.edu/doi/10.1086/626801

19. Raith, M.M. Raase, P., Reinhardt, J. Guide to Thin Section Microscopy. 2nd ed. Mineral Society of America; 2012.

20. Sørensen BE. A revised Michel-Lévy interference colour chart based on first-principles calculations. Eur J Mineral. 2013 Feb 11;25(1):5–10.

21. Davidson MW. Evident Scientific (Olympus Scientific Solutions). [cited 2025 Feb 7]. The First Order (Full Wave) Retardation Plate. Available from: https://evidentscientific.com/en/microscope-resource/knowledge-hub/techniques/polarized/firstorderplate

22. Maschalchi P. Github. [cited 2025 Feb 7]. White balance correction. Available from: https://github.com/pmascalchi/ImageJ_Auto-white-balance-correction

23. Budke C, Dreyer A, Jaeger J, Gimpel K, Berkemeier T, Bonin AS, et al. Quantitative efficacy classification of ice recrystallization inhibition agents. Cryst Growth Des. 2014 Sept 3;14(9):4285–94.

24. Urbańczyk M, Góra J, Latajka R, Sewald N. Antifreeze glycopeptides: from structure and activity studies to current approaches in chemical synthesis. Amino Acids. 2017 Feb;49(2):209–22.
